# The Human Cytomegalovirus Nonstructural Glycoprotein UL148 Reorganizes the Endoplasmic Reticulum

**DOI:** 10.1128/mBio.02110-19

**Published:** 2019-12-10

**Authors:** Hongbo Zhang, Clarissa Read, Christopher C. Nguyen, Mohammed N. A. Siddiquey, Chaowei Shang, Cameron M. Hall, Jens von Einem, Jeremy P. Kamil

**Affiliations:** a Department of Microbiology and Immunology, LSU Health Sciences Center, Shreveport, Louisiana, USA; b Center for Molecular and Tumor Virology, LSU Health Sciences Center, Shreveport, Louisiana, USA; c Research Core Facility, LSU Health Sciences Center, Shreveport, Louisiana, USA; d Institute of Virology, Ulm University Medical Center, Ulm, Germany; e Central Facility for Electron Microscopy, Ulm University, Ulm, Germany; Princeton University

**Keywords:** cytomegalovirus, glycoproteins, herpesviruses, organelle structure, secretory pathway, stress adaptation, stress response, unfolded protein response, virus entry

## Abstract

Human cytomegalovirus (HCMV) encodes an endoplasmic reticulum (ER)-resident glycoprotein, UL148, which activates the unfolded protein response (UPR) but is fully dispensable for viral replication in cultured cells. Hence, its previously ascribed roles in immune evasion and modulation of viral cell tropism are hypothesized to cause ER stress. Here, we show that UL148 is necessary and sufficient to drive the formation of prominent ER-derived structures that on average occupy 5% of the infected cell cytoplasm. The structures are sites where UL148 coalesces with cellular proteins involved in ER quality control, such as HRD1 and EDEM1. Electron microscopy revealed that cells infected with wild-type but not *UL148*-null HCMV show prominent accumulations of densely packed ruffled ER membranes which connect to distended cisternae of smooth and partially rough ER. During ectopic expression of UL148-green fluorescent protein (GFP) fusion protein, punctate signals traffic to accumulate at conspicuous structures. The structures exhibit poor recovery of fluorescence after photobleaching, which suggests that their contents are poorly mobile and do not efficiently exchange with the rest of the ER. Small-molecule blockade of the integrated stress response (ISR) prevents the formation of puncta, leading to a uniform reticular fluorescent signal. Accordingly, ISR inhibition during HCMV infection abolishes the coalescence of UL148 and HRD1 into discrete structures, which argues that UL148 requires the ISR to cause ER reorganization. Given that UL148 stabilizes immature forms of a receptor binding subunit for a viral envelope glycoprotein complex important for HCMV infectivity, our results imply that stress-dependent ER remodeling contributes to viral cell tropism.

## Abstract (precis)

Perturbations to endoplasmic reticulum (ER) morphology occur during infection with various intracellular pathogens and in certain genetic disorders. We identify that a human cytomegalovirus (HCMV) gene product, UL148, profoundly reorganizes the ER during infection and is sufficient to do so when expressed on its own. Our results reveal that UL148-dependent reorganization of the ER is a prominent feature of HCMV-infected cells. Moreover, we find that this example of virally induced organelle remodeling requires the integrated stress response (ISR), a stress adaptation pathway that contributes to a number of disease states. Since ER reorganization accompanies roles of UL148 in modulation of HCMV cell tropism and in evasion of antiviral immune responses, our results may have implications for understanding the mechanisms involved. Furthermore, our findings provide a basis to utilize UL148 as a tool to investigate organelle responses to stress and to identify novel drugs targeting the ISR.

## INTRODUCTION

UL148 is a human cytomegalovirus (HCMV) endoplasmic reticulum (ER)-resident glycoprotein that plays roles in the evasion of cell-mediated immunity and shows intriguing effects on cell tropism. During infection of epithelial cells, viruses disrupted for *UL148* replicate to produce roughly 100-fold enhanced levels of infectious progeny virions compared to that of the wild type (1). These effects correlate with reduced expression of glycoprotein O (gO), a subunit of a heterotrimeric viral glycoprotein H (gH)/glycoprotein L (gL) complex (gH/gL/gO) that is required for the infectivity of cell-free virions (2–4). The presence of gO in the context of the heterotrimer endows the virus with the capacity to utilize the platelet-derived growth factor receptor α (PDGFRα) as an entry receptor (5–7). Accordingly, UL148 has been found to stabilize immature forms of gO prior to their assembly into gH/gL/gO heterotrimers (1, 8). Despite that UL148 does not stably associate with gO, the data suggest an interaction with gH (1).

UL148 also physically associates with CD58 (LFA-3), a costimulatory ligand for natural killer cells and T lymphocytes, preventing its presentation at the cell surface (9). Although the mechanisms by which UL148 stabilizes gO and retains CD58 within the ER remain unknown, UL148 strongly contributes to activation of the unfolded protein response (UPR) during infection and is sufficient to activate the UPR when ectopically expressed in noninfected cells (10). UL148 copurifies from infected cells with SEL1L, an adaptor subunit of ER-based E3 ubiquitin ligase HRD1 (SYVN1) that plays important roles in ER-associated degradation (ERAD) of terminally misfolded glycoproteins (8). This suggests a physical interaction with the ERAD machinery, which may be germane to the mechanism by which UL148 activates the UPR.

Here, we show that UL148 is necessary and sufficient to induce unusual ER structures at which large quantities of ER factors involved in glycoprotein quality control accumulate. Electron microscopy analyses show that the UL148-induced structures are composed of densely packed and ruffled membranes, most likely representing collapsed ER, that connect to ER tubules of highly distended cisternal space. Furthermore, data from inhibitor studies strongly argue that ER remodeling triggered by UL148 requires the integrated stress response. Overall, our results reveal a striking ER perturbation induced by HCMV which is entirely controlled by a single viral gene product. These findings may have important implications for understanding how UL148 regulates viral cell tropism and contributes to evasion of host immune defenses.

## RESULTS

### UL148 causes reorganization of ER quality control proteins into unusual globular structures.

**FIG 1:**
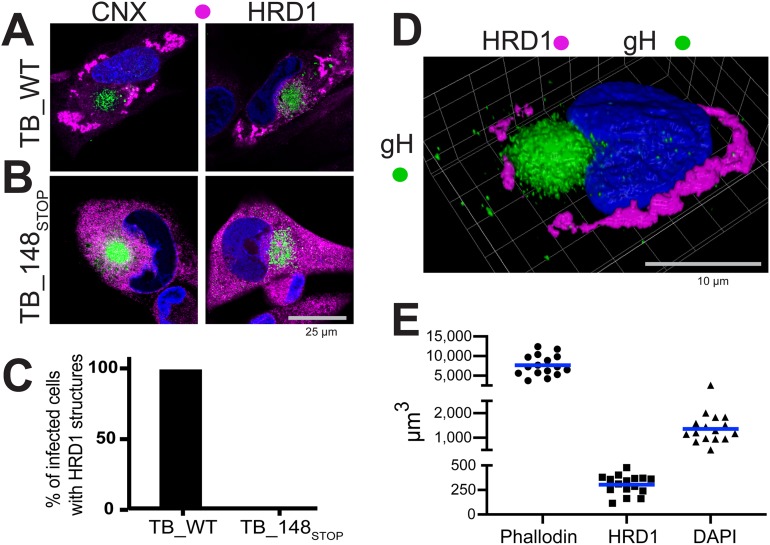
UL148 reorganizes ER markers into anomalous structures during HCMV infection. Fibroblasts infected at an MOI of 1 with either wild-type HCMV strain TB40/E (TB_WT) (A) or an isogenic *UL148*-null mutant (TB_148_STOP_) (B) were fixed 96 h postinfection (hpi) and imaged by confocal microscopy after staining with antibodies specific for calnexin (CNX) or HRD1 (magenta) and glycoprotein H (gH; green), as indicated. DAPI (blue) signal was used to counterstain nuclei. (C) Percentages of fibroblasts that contain HRD1 structures at 96 hpi; 50 gH-positive cells for each condition were scored for the presence or absence of UL148 structures (also see Fig. S1A in the supplemental material). (D) 3D confocal image projection of a TB_WT-infected fibroblast stained at 96 hpi for HRD1 and gH. (E) Volumetric measurements were made from 16 infected cells, stained with phalloidin-Alexa Fluor 594 conjugate to estimate total cell volume, with HRD1 antibody to estimate the volume of UL148-dependent ER structures, and with DAPI to estimate nuclear volume.

UL148 was previously observed to colocalize with the ER marker calnexin during infection (1). Nonetheless, HCMV-infected cells did not show the uniform reticular calnexin staining pattern characteristic for the ER marker in uninfected cells. We later noticed that cells infected with a *UL148*-null virus showed uniform calnexin staining (see below). To formally determine whether UL148 influences calnexin localization, we compared fibroblasts at 4 days postinfection (dpi) with either wild-type strain TB40/E virus (TB_WT) or a *UL148*-null mutant (TB_148_STOP_) (8, 10), each derived from an infectious bacterial artificial chromosome (BAC) clone of HCMV strain TB40/E (11) (Fig. 1). In cells infected with wild-type virus, calnexin antibodies stained unusual globular structures at the cell periphery, as expected (1) (Fig. 1A and C; see also Fig. S1A in the supplemental material). However, in cells infected with the *UL148*-null virus, calnexin staining was uniform throughout the cytoplasm (Fig. 1B and C), as would be expected for an ER marker in uninfected cells. The staining pattern for another ER marker, HRD1, likewise indicated accumulation at unusual globular structures during wild-type HCMV infection but not during infection with *UL148*-null mutant viruses (Fig. 1; Fig. S1A). Because *UL148* is fully dispensable for efficient viral replication in fibroblasts (1), these results suggest that redistribution of the two ER markers depends on UL148.

10.1128/mBio.02110-19.1 FIG S1 Additional indirect immunofluorescent confocal microscopy results from infected cells. Note that unless otherwise indicated, cells were fixed and stained at 96 h postinfection (hpi). (A) Lower magnification overview of results shown in Fig. 1A and B; staining of HCMV glycoprotein H (gH) and HRD1 in human fibroblasts infected with wild-type HCMV strain TB40/E (WT) or the *UL148*-null derivative TB_148_STOP_. (B) gH and HRD1 staining of human fibroblasts infected with wild-type Merlin recovered from BAC-cloned Merlin pAL1393 (ME_WT) or a *UL148*-null derivative of the same virus (ME_148_STOP_). (C) gH and HRD1 staining of human fibroblasts infected with wild-type HCMV strain TRgfp recovered from BAC-cloned TRgfp (TR_WT) or a *UL148*-null derivative of the same virus (TR_148_STOP_). (D) Staining of calnexin and the viral tegument protein UL71 in human fibroblasts fixed 5 days following infection with primary clinical HCMV isolates obtained from patient throat swabs. (E) HA and HRD1 staining in THP-1 macrophages infected with HCMV strain TB40/E derivative viruses TB_148^HA^ or TB_159^HA^; note that THP-1 monocytes were differentiated using phorbol ester treatment prior to infection. (F) Staining of HA and HRD1 in ARPE-19 epithelial cells infected with either TB_148^HA^ or TB_159^HA^. (G) HA and HRD1 staining of ARPE-19 cells infected with HCMV strain AD169 repaired for *UL131* and to which an HA-tagged UL148 from strain TB40/E was restored at the native *UL148* locus (AD^r131^_148^HA^) or with a parental AD169 virus repaired for *UL131* to which *UL148* was not restored (AD^r131^). Note that (i) a functional *UL131* is required for efficient infection of epithelial cells; (ii) for ADr131, we did not employ a viral marker (e.g., HA) to identify infected cells, and so the appearance of syncytia and the characteristic kidney bean- shaped nucleus was used to indicate infected cells and a slightly lower magnification was used to best show these features. Hence, a different scale bar was used. (H) HA and HRD1 staining in telomerase-immortalized rhesus fibroblasts infected with BAC-derived rhesus CMV (RhCMV) strain 68-1 carrying an HA-tag at the C terminus of Rh159. (I) LC3B and syntaxin 6 (STX6) staining in human fibroblasts infected with the indicated viruses. Download FIG S1, PDF file, 0.9 MB. Copyright © 2019 Zhang et al. 2019 Zhang et al. This content is distributed under the terms of the Creative Commons Attribution 4.0 International license.

As expected (12, 13), antibodies specific for the viral envelope glycoprotein glycoprotein H (gH) stained a juxtanuclear compartment, termed the cytoplasmic virion assembly compartment (cVAC), which does not involve the ER (Fig. 1). Similarly, contrasting staining patterns for HRD1 and/or calnexin were observed in cells infected with wild-type and *UL148*-null mutants of clinical HCMV strains Merlin and TR (Fig. S1B to D). Reciprocally, we restored a functional *UL148* at its native locus in the context HCMV strain AD169, which spontaneously lost most of the gene, along with much of the *ULb′* region, during serial passage in cultured cells (14). In cells infected with AD169 repaired for *UL148*, but not the *UL148*-null parental virus, the HRD1 staining pattern showed prominent globular structures (Fig. S1G).

Because similar differences in HRD1 staining were also seen during wild-type versus *UL148*-null infections of THP-1 macrophages and ARPE-19 epithelial cells (Fig. S1E to G), we conclude that UL148-dependent reorganization of ER markers occurs in multiple cell types. Moreover, the calnexin staining pattern seen with four different primary clinical isolates from patient throat swabs likewise indicated punctate globular structures (Fig. S1D). These observations, taken together with results from formal comparisons of wild-type versus *UL148*-null mutants of four different BAC-cloned HCMV strains (Fig. 1; Fig. S1), argue that UL148 profoundly affects the ER during infection.

We measured the three-dimensional volume of UL148-dependent HRD1 structures from 16 cells fixed at 96 h postinfection (hpi) with strain TB40/E. On average, the structures occupied 303.3 μm^3^ (standard error of the mean [SEM], ±24.8 μm^3^) of a total cell volume of 7,657 μm^3^ (SEM, ±651.0 μm^3^), which was estimated using a phalloidin-fluorophore conjugate to detect the actin cytoskeleton (Fig. 1D and E). Subtracting the volume of nuclei, as indicated by 4′,6-diamidino-2-phenylindole (DAPI) (average nucleus, 1,360 μm^3^; SEM, ±129.4 μm^3^), we calculate that, on average, the structures occupy 5.2% of the cytosolic volume (SEM, ±0.61%; range, 2.0% to 7.0%). Based on these findings, taken together with our results from additional wild-type (WT) versus *UL148*-null mutant HCMV strains (Fig. S1B to G), we conclude that the UL148-dependent ER structures are a prominent feature of HCMV-infected cells.

UL148 contributes to activation of the unfolded protein response (UPR) during HCMV infection (10) and copurifies from infected cells with SEL1L (8), an adaptor subunit for the E3 ubiquitin ligase HRD1, which plays crucial roles in ER-associated degradation (ERAD) of terminally misfolded glycoprotein substrates (15). Hence, the accumulation of HRD1 and calnexin at unusual structures during wild-type but not *UL148*-null infection may suggest that the structures form in response to defects in ER quality control (ERQC) caused by UL148. Indeed, the overexpression of terminally misfolded glycoproteins during proteasomal inhibition causes cellular factors involved in ERQC, such as calnexin, to compartmentalize from the rest of the ER, while other ER markers such as BiP (GRP78) or protein disulfide isomerase (PDI) remain largely unaltered (16–18).

To gain further insights into the nature of these peculiar ER structures, we set out to develop a more comprehensive understanding of their protein composition. We infected fibroblasts with TB_148^HA^ (1), an HCMV strain TB40/E derivative that expresses a hemagglutinin (HA) epitope tag at the C terminus of UL148, or TB_159^HA^, a *UL148*-null comparator that expresses *Rh159* instead of UL148, and stained for various ER markers at 96 hpi.

**FIG 2:**
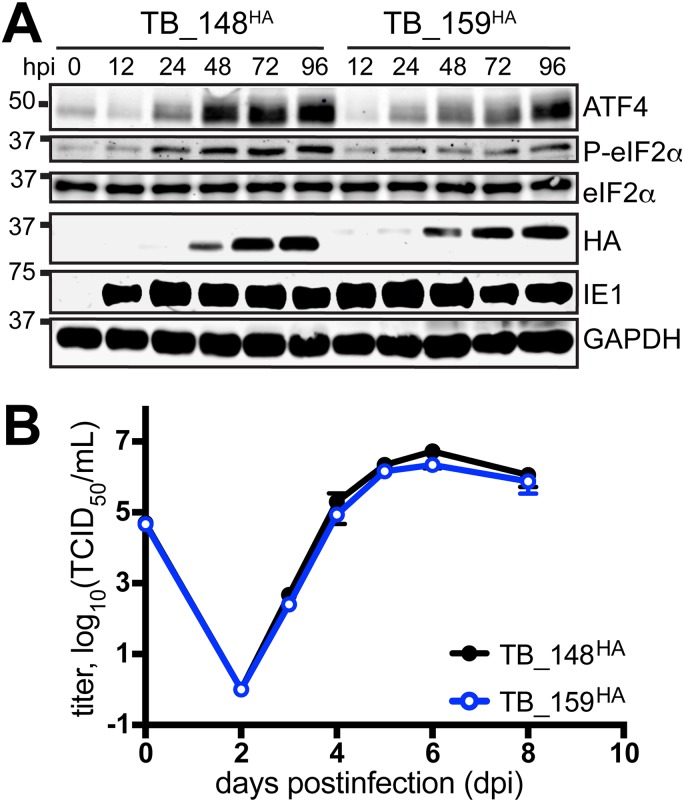
Characterization of TB_148^HA^ and TB_159^HA^ viruses. (A) Human fibroblasts were infected at an MOI of 1 with the indicated recombinant HCMVs. Cell lysate samples harvested at the indicated times postinfection (h postinfection [hpi]) were analyzed by Western blotting to detect HA-tagged UL148 or Rh159, the 72-kDa viral nuclear antigen IE1-72 (IE1), and GAPDH. (B) Single-cycle viral replication kinetic curves from fibroblasts infected at an MOI of 1 were plotted by determining the titer in 50% tissue culture infectious dose (TCID_50_) from supernatants collected at the indicated times postinfection.

We considered the TB_159^HA^ virus to be an appropriate control for the following reasons. First, Rh159 and UL148 exhibit ∼30% identity at the amino acid level, and both glycoproteins localize to the ER and block cell surface presentation of immune cell-activating ligands; UL148 retains CD58, a ligand for CD2, while Rh159 retains NKG2D ligands of the MIC and ULBP families (1, 9, 19). Second, Rh159 is expressed from TB_159^HA^ at comparable levels and with kinetics similar to those observed for UL148 from TB_148^HA^, and the two viruses replicate indistinguishably in fibroblasts (Fig. 2). Moreover, consistent with results from ectopic expression studies (10), Rh159 does not appear to activate the UPR to the same extent as UL148, as evidenced by lower levels of phospho-eIF2α and ATF4 during TB_159^HA^ infection (Fig. 2A).

**FIG 3:**
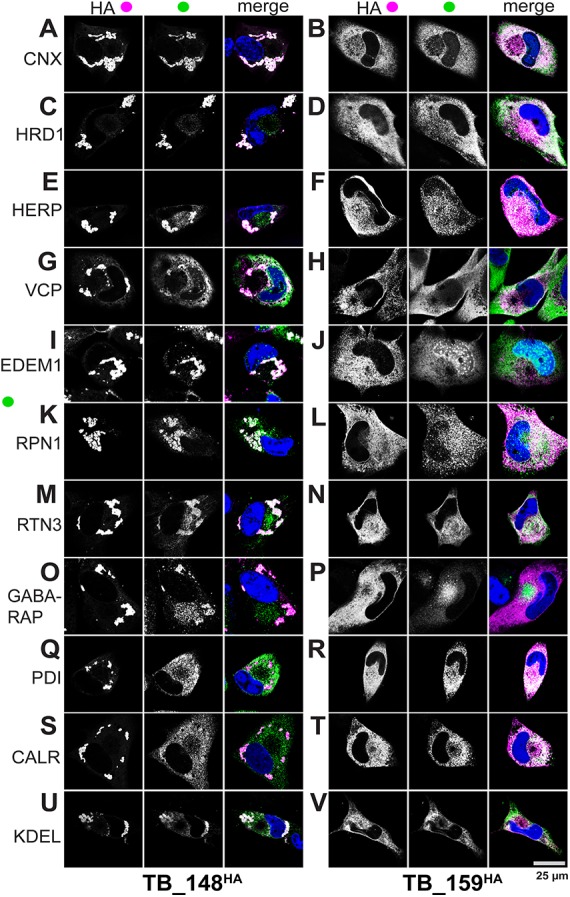
UL148 localizes to unusual ER compartments that are enriched for glycoprotein quality control markers. Fibroblasts infected at an MOI of 1 with either TB_148^HA^ (A, C, E, G, I, K, M, O, Q, S, and U) or TB_ 159^HA^ (B, D, F, H, J, L, N, P, R, T, and V) were fixed 96 h postinfection and imaged by confocal microscopy after costaining with antibodies specific for HA (UL148/Rh159, magenta in merge) and the indicated cellular markers (green in merge). DAPI (blue) counterstaining is shown in merged images.

ERQC markers failed to coalesce into unusual structures during infection with TB_159^HA^ (Fig. 3). The structures likewise failed to occur in rhesus fibroblasts during infection with a recombinant rhesus cytomegalovirus (RhCMV) that expresses HA-tagged Rh159 (Fig. S1H). The latter argues against the possibility that Rh159 requires the context of rhesus cells to redistribute ER markers in a manner analogous to what is seen for UL148 during HCMV infection.

**FIG 4:**
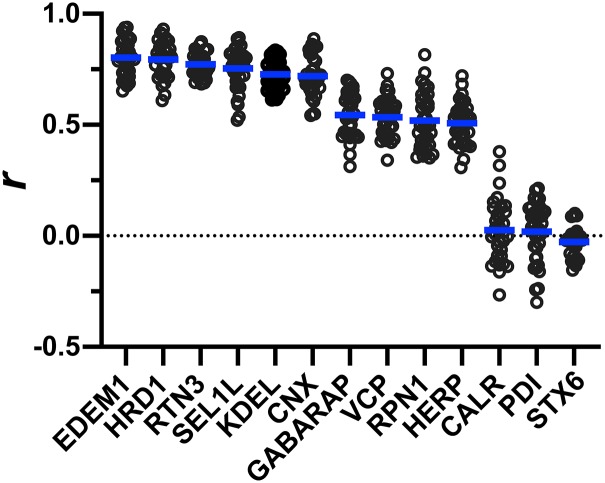
Quantification of colocalization between UL148 and cellular markers. A Pearson’s correlation coefficient (*r*) was calculated using NIH ImageJ software to estimate the degree of colocalization between UL148 (HA signal) and each of the indicated cellular markers. A minimum of 30 cells were analyzed per marker. The arithmetic mean for each colocalization analysis result is shown as a blue line, and data points for individual cells analyzed are plotted as circles.

In cells infected with TB_148^HA^, HA antibody staining indicated localization of UL148 to globular structures, as expected (1). Antibody signals from indirect confocal immunofluorescence detection of cellular ER resident proteins involved in ERQC, including calnexin (CNX), HRD1, SEL1L, HERPUD1 (HERP), valosin-containing protein (VCP, p97), and EDEM1, strongly colocalized with signals from HA-tagged UL148 (Fig. 3 and 4). In contrast, we observed uniform ER staining patterns for PDI and calreticulin (CALR), indicating that these ER markers do not localize to the UL148 structures (Fig. 3Q and S). Intriguingly, antibody signals detecting reticulon 3 (RTN3) and ribophorin 1 (RPN1), which are markers for smooth ER and rough ER, respectively, each appreciably colocalized with the UL148 (HA) signal at the induced structures (Fig. 3K and M), which indicates that the structures may involve both rough and smooth ER. LC3B failed to colocalize with the structures (Fig. S2), as might be expected given that the virus inhibits macroautophagy at late times during infection (20, 21). Nevertheless, our results may suggest the presence of a related mammalian ATG8 ortholog, GABARAP (Fig. 3O).

10.1128/mBio.02110-19.2 FIG S2 Cellular proteins involved in glycoprotein quality control are recruited with differing kinetics to UL148 ER structures. Fibroblasts infected with TB_148^HA^ at an MOI of 1 were fixed at the indicated time points (days postinfection [dpi]) and imaged by confocal microscopy after staining with antibodies specific for HA (UL148, magenta) and CNX (green) (A), HRD1 (green) (B), or VCP (green (C); DAPI staining is in blue. Download FIG S2, TIF file, 2.3 MB. Copyright © 2019 Zhang et al. 2019 Zhang et al. This content is distributed under the terms of the Creative Commons Attribution 4.0 International license.

In cells infected with TB_159^HA^, all of the ER markers we examined showed uniform reticular staining, as did the anti-HA signal detecting Rh159 (Fig. 3B, D, F, H, J, L, N, P, R, and T). EDEM1, in addition to showing reticular staining, also labeled puncta associated with the nucleus (Fig. 3J and I), which may represent enriched levels of the protein at the rough ER membranes associated with the nuclear envelope, although we cannot exclude the possibility of spurious intranuclear staining. GABARAP antibodies stained the cVAC in TB_159^HA^-infected cells but also showed a much weaker diffuse signal throughout the cytosol. Even though we took steps to block viral Fc receptors, which can cause rabbit antibodies to nonspecifically label the cVAC (22), it is plausible that GABARAP antibody signal from the cVAC reflects incomplete blocking of viral Fc receptors. For both viruses, antibodies specific for the *trans*-Golgi network (TGN) marker syntaxin 6 (STX6) and gH, as expected, stained the juxtanuclear cVAC structure (12, 13, 23) (Fig. S1I).

To quantify the degree of colocalization with UL148, we calculated Pearson’s correlation coefficients from a minimum of 30 TB_148^HA^-infected cells per staining condition, comparing the overlap of signal from each ER marker with HA signal from UL148. The correlation coefficients (*r*) for staining patterns from antibodies specific for EDEM1, HRD1, reticulon 3 (RTN3), SEL1L, the ER retention motif KDEL (EKDEL), and calnexin ranged from 0.8 to 0.72. These results suggest that these proteins, and certain lumenal ER resident proteins that contain a C-terminal KDEL motif in the context EKDEL, such as BiP (GRP78) and GRP94, extensively colocalize with UL148 at the induced structures (Fig. 4). Meanwhile, GABARAP, VCP, ribophorin-1 (RPN1), and HERP showed *r* values in the range of 0.54 to 0.51, indicating a moderate colocalization with UL148. However, CALR and PDI, which, like BiP, are lumenal ER residents that carry KDEL motifs (though not in the context EKDEL), and the TGN marker syntaxin 6, gave *r* values of close to zero, indicating that these markers do not appreciably colocalize with UL148, as is consistent with our immunofluorescence data suggesting that these markers negligibly associate with the unusual structures. From these results, we conclude that the UL148-dependent ER structures are enriched for cellular markers involved in glycoprotein quality control. In this regard, the structures resemble the “ER quality control (ERQC) compartments” described by Lederkremer and colleagues (16, 17, 24).

### VCP and HRD1 are recruited to incipient UL148 ER structures prior to calnexin.

To determine whether there might be differences in the kinetics of recruitment of ERQC markers during the formation of ER structures, we examined a series of time points from 1 to 4 days postinfection (dpi) with TB_148^HA^, staining for three different ERQC markers, calnexin, HRD1, and VCP, alongside UL148 (HA). At 1 dpi, the signal from UL148 was only faintly detected, as expected (1), while each of the ER markers was readily detected, providing a readout of their staining patterns prior to being substantially perturbed by UL148 (see Fig. S2). At 2 dpi, we detected robust anti-HA signal, indicating the presence of UL148. At this time point, UL148 exhibited intense signal at small globular puncta, which we interpret to represent incipient UL148 structures, as well as more diffuse staining of a reticular structure consistent with undisturbed ER. Calnexin did not appreciably colocalize with the UL148 puncta until at least 3 dpi, and the structures were not readily visualized by calnexin staining until 4 dpi (Fig. S2). In contrast, signals from HRD1 and VCP staining were sufficient to mark the UL148 puncta by 2 dpi. Notably, the appearance of HRD1 at the structures prior to that of calnexin is consistent with our previous results showing that UL148 copurifies from infected cells with SEL1L, an adaptor subunit for HRD1 (8). These results imply that basal elements of the ERAD machinery, exemplified by HRD1 and VCP, may be recruited to the UL148 ER structures prior to calnexin.

### Visualization of UL148-induced ER structures by electron microscopy.

**FIG 5:**
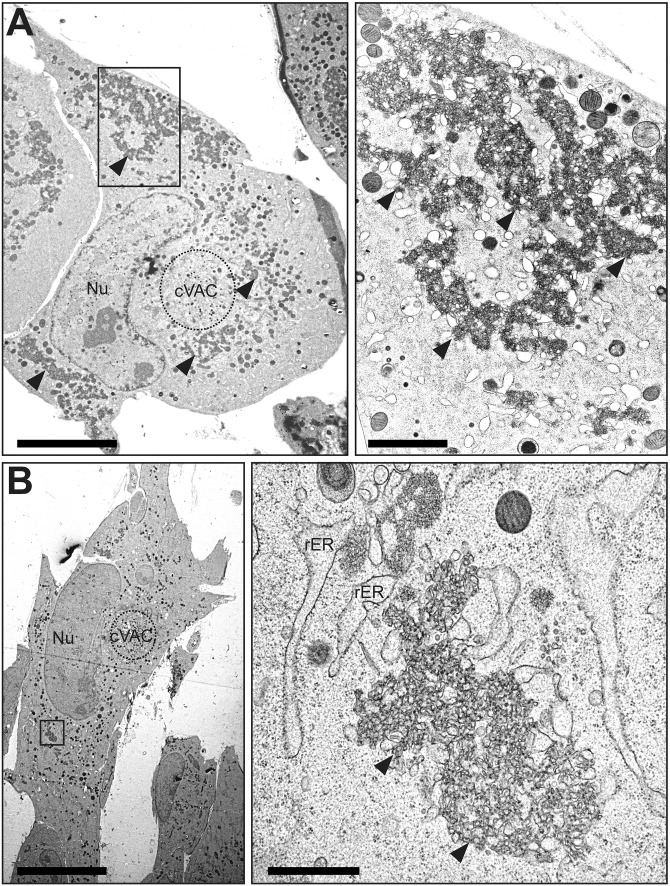
TEM of ER structures in wild-type HCMV-infected cells. Human fibroblasts infected with wild-type HCMV (TB_WT) were fixed by high-pressure freezing and freeze substitution at day 5 postinfection and imaged using TEM. (A and B) Cell overviews at left. For each cell, the boxed region is shown at higher magnification. Scale bars, 10 μm (left), 2 μm (right); rER, rough ER; Nu, nucleus; cVAC, cytoplasmic viral assembly compartment. Solid arrowheads indicate the UL148-dependent ER structures of interest.

**FIG 6:**
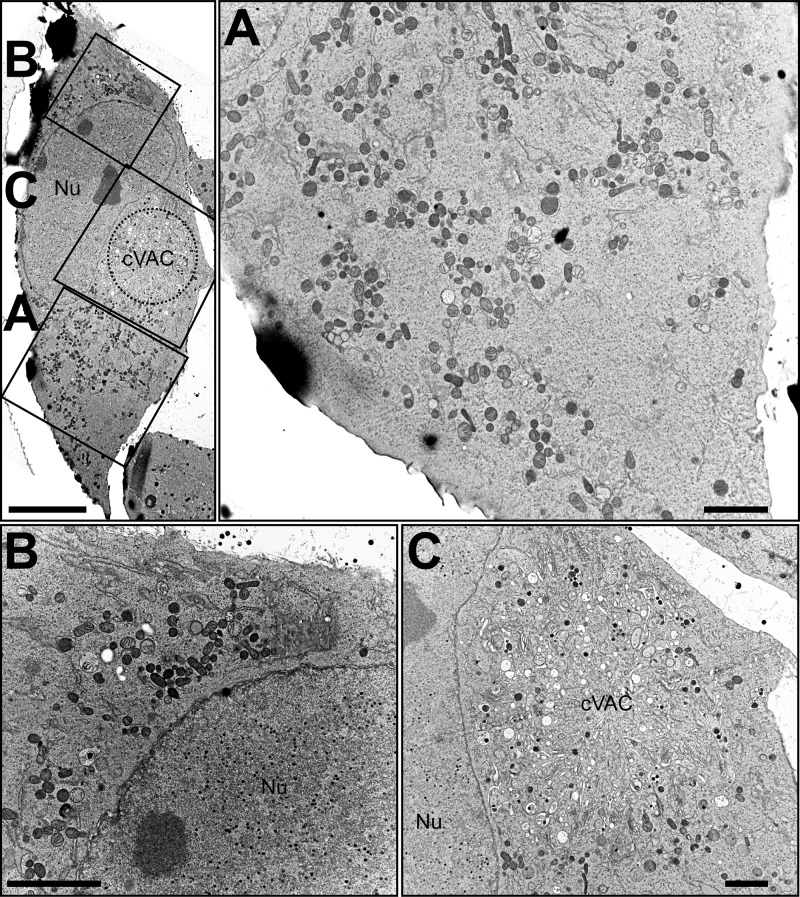
TEM of *UL148*-null HCMV-infected cells. Human fibroblasts infected with a *UL148*-null mutant (TB_148_STOP_) were fixed by high-pressure freezing and freeze substitution at day 5 postinfection and imaged using TEM. (Top left) Overview of a representative cell shown in panels A, B, and C (each boxed). The boxed regions are expanded at higher magnification at right (A) and at the bottom (B and C). Scale bars, 10 μm (top left), 2 μm (panels A to C). Nu, nucleus; cVAC, cytoplasmic viral assembly compartment.

**FIG 7:**
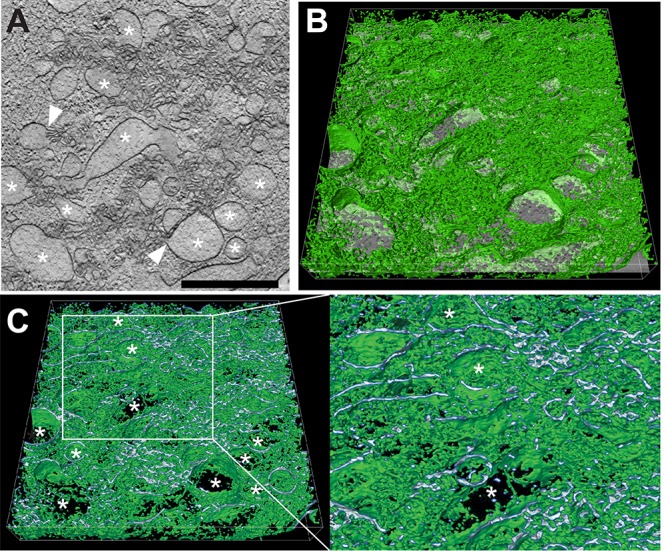
STEM tomography of UL148-dependent ER structures in HCMV-infected cells. Human fibroblasts infected with wild-type HCMV (TB_WT) were fixed by high-pressure freezing and freeze substitution at day 5 postinfection, and tomograms were recorded by STEM. (A) Virtual section through the tomogram of a virus-induced membranous structure; *, ER structures of distended lumenal space. White arrowheads indicate sites at which membranes originating from distended ER cisternae continue into areas of involuted collapsed ER. Scale bar, 1 μm. (B) Same virtual section as in panel A tilted and with a 3D visualization of the membranous network (green) of the entire tomogram.*, the same distended ER cisternae as in panel A. (C) Cross section through image shown in panel B to visualize the membrane profile of the membranous structures. The region delimited by a white box is shown at higher magnification on the right. Finer detail of the enlarged ER cisternae and the connections between them are readily visible; *, the same distended ER cisternae as in panels A and B. Scale bar, 1 μm.

To discern the ultrastructural appearance of the UL148 structures and to formally ascertain their relationship to the ER, we carried out transmission electron microscopy (TEM) imaging of wild-type (TB_WT)- and *UL148*-null (TB_148_STOP_)-infected fibroblasts at 5 dpi. In high-pressure frozen and freeze-substituted infected fibroblasts, cells with a high density of viral nucleocapsids within the nucleus were selected for analysis, as this feature indicates late time points during infection when UL148 is abundantly expressed. Consistent with our confocal immunofluorescence data, TEM results revealed prominent globular and oblong membrane accumulations in the cytoplasm of wild-type virus-infected fibroblasts but not in *UL148*-null-infected controls (Fig. 5 and 6). These structures stand out for their high electron density and were found in areas of the cytoplasm in TB_WT-infected cells that were not occupied by the cVAC, correlating with the localization of ERQC markers and UL148 by fluorescence microscopy. In TEM, these electron-dense areas were characterized by accumulations of densely packed ruffled membranes. Moreover, the ruffled membrane accumulations were associated with smooth and partially rough ER structures of seemingly enlarged cisternal space, suggesting that the ruffled membranes derive from collapsed ER membranes. Scanning transmission electron microscopy (STEM) tomography confirmed the highly complex membranous meshwork of the HCMV-induced ER structures at high resolution in three dimensions (Fig. 7). Tracking of individual membranes revealed the ruffled ER membranes as interconnected throughout the volume of the tomogram and most likely throughout the globular structure (Fig. 7B and C). Furthermore, the tomogram shows that the ruffled membranes are continuous with enlarged ER cisternae and thus likely originated from the ER.

### UL148 accumulates in a detergent-insoluble form during infection.

Disease-associated variants of certain cellular proteins, such as the A103E mutant of the calcium channel ORAI1 (A103E ORAI1), and the E342K “Z” variant of alpha 1 antitrypsin (Z A1AT), localize to anomalous ER structures reminiscent of those we observe to depend on UL148 (18, 25–28). Z A1AT and A103E ORAI1 accumulate in detergent-insoluble forms within the ER and within ER membranes, respectively, which indicates aggregation or polymerization of the proteins and suggests a mechanism that may contribute to the formation of ER structures (18, 25, 28, 29). We therefore set out to determine whether differences in solubility might correlate with the differing potentials of UL148 and Rh159 to cause ER reorganization and to activate the UPR (10). To address this question, we infected fibroblasts with TB_148^HA^ or TB_159^HA^ at a multiplicity of infection (MOI) of 1 and, at various times postinfection, prepared cell lysates in radioimmunoprecipitation assay (RIPA) buffer. After subjecting the lysates to high-speed centrifugation, we examined the relative levels of UL148 and Rh159 in the detergent-soluble supernatant and detergent-insoluble pellet fractions.

**FIG 8:**
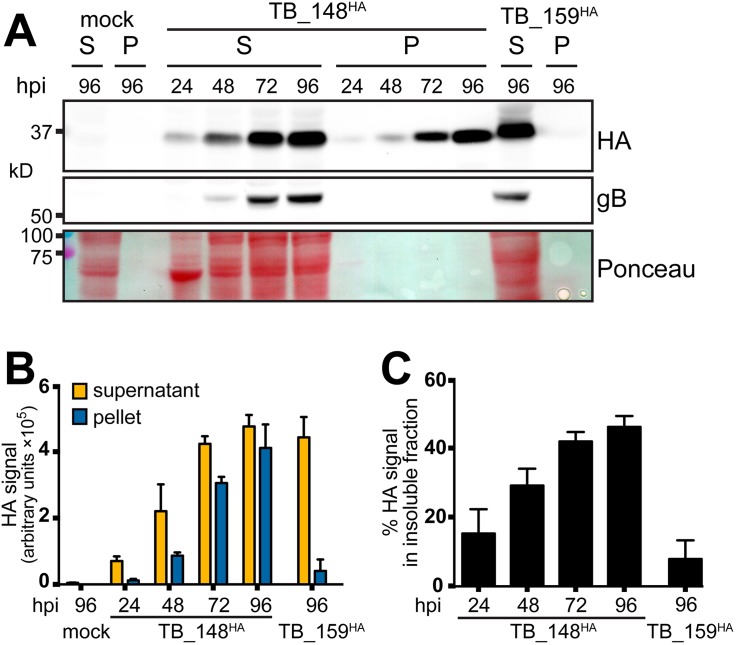
Solubility analysis of UL148 and Rh159. (A) Human fibroblasts were infected at an MOI of 1 TCID_50_/cell with TB_148^HA^, HCMV strain TB40/E-derived virus that expresses UL148 fused to a C-terminal HA tag, or TB_159^HA^, which lacks *UL148* and instead expresses rhesus CMV Rh159 carrying a C-terminal HA tag. At the indicated times postinfection (hpi), infected cells were collected in radioimmunoprecipitation (RIPA) lysis buffer and centrifuged at 21,000 × *g* for 30 min, after which supernatant (S) and pellet (P) fractions were boiled in gel loading buffer containing 2% sodium dodecyl sulfate (SDS). Equivalent portions of supernatant and pellet were resolved by SDS-PAGE and transferred to a nitrocellulose membrane for detection of protein species immunoreactive to antibodies against HA and gB and for total protein signal using Ponceau S reagent (Ponceau). (B and C) Signal intensity of fluorophore-conjugated secondary antibodies in anti-HA Western blots were measured from three independent biological replicates of the experiment shown in panel A; error bars indicate standard deviations. (B) The fluorescent signal for each infection time point condition (*y* axis is in arbitrary units, in hundred thousands). (C) The amount of signal found in the insoluble (pellet) fraction relative to the total signal (pellet plus supernatant) for each infection time point is plotted as a percentage value.

A substantial portion of UL148 was detected from the detergent-insoluble fractions at all time points tested (Fig. 8). Furthermore, the percentage of UL148 detected within the insoluble fraction increased over time. In contrast, Rh159 was found only in the detergent-soluble fraction. From these results, we conclude that UL148 but not Rh159 accumulates in a detergent-insoluble form during infection. Because the anti-HA immunoreactive band detected in both the soluble and insoluble fractions from TB_148^HA^-infected cells showed a relative mobility of ∼35 kDa, which matches that expected for the mature endoH-sensitive glycoprotein (1), these findings argue that UL148 may form aggregates or polymers within the ER.

### UL148 is sufficient to compartmentalize the ER.

Our data thus far demonstrate that UL148 is necessary during infection to cause redistribution of cellular ER markers for glycoprotein quality control processes, such that a substantial portion of the ERQC machinery appears to become sequestered away from the rest of the organelle into novel membranous structures. Because a number of viral proteins that remodel the ER during infection are sufficient to alter ER morphology when ectopically expressed (e.g., see references 30 and 31, reviewed in reference 32), we wondered whether UL148 expression would be sufficient to induce ER structures akin to those seen during HCMV infection. Making use of “tet-on” ARPE-19 epithelial cells that inducibly express either UL148 or Rh159, each carrying a C-terminal HA-tag (10), we induced transgene expression for 48 h and subsequently stained for various cellular markers of the ER for ERQC, as well as for the ATG8 family proteins LC3B and GABARAP, which play roles in macroautophagy and related processes.

In cells expressing UL148 (i148^HA^), we observed that calnexin, HRD1, EDEM1, and VCP colocalized with UL148 at prominent globular structures reminiscent of those observed during infection (see Fig. S3). The respective rough and smooth ER markers ribophorin 1 (RPN1) and reticulon 3 (RTN3), as well as the ATG8 proteins LC3B and GABARAP, likewise colocalized to the UL148-induced structures. However, PDI and CALR did not do so (Fig. S3) or, at best, showed only limited colocalization, consistent with our results from infected cells (Fig. 3 and 4). Signals from antibodies specific for the KDEL (EKDEL) motif important for ER retrieval of luminal ER residents, such as BiP and GRP94, likewise showed only limited colocalization with UL148. The PDI and KDEL results suggest that large regions of the ER are not involved in the UL148 structures.

10.1128/mBio.02110-19.3 FIG S3 UL148 is sufficient to remodel the ER. Expression of HA-tagged UL148 or Rh159 was induced in tet-on ARPE-19 epithelial cells, i148^HA^ or i159^HA^, respectively. Cells were fixed 48 h following doxycycline induction and processed for indirect immunofluorescence staining to detect the indicated cellular markers (green) together with HA (magenta). Scale bar, 10 μm. Download FIG S3, PDF file, 0.9 MB. Copyright © 2019 Zhang et al. 2019 Zhang et al. This content is distributed under the terms of the Creative Commons Attribution 4.0 International license.

**FIG 9:**
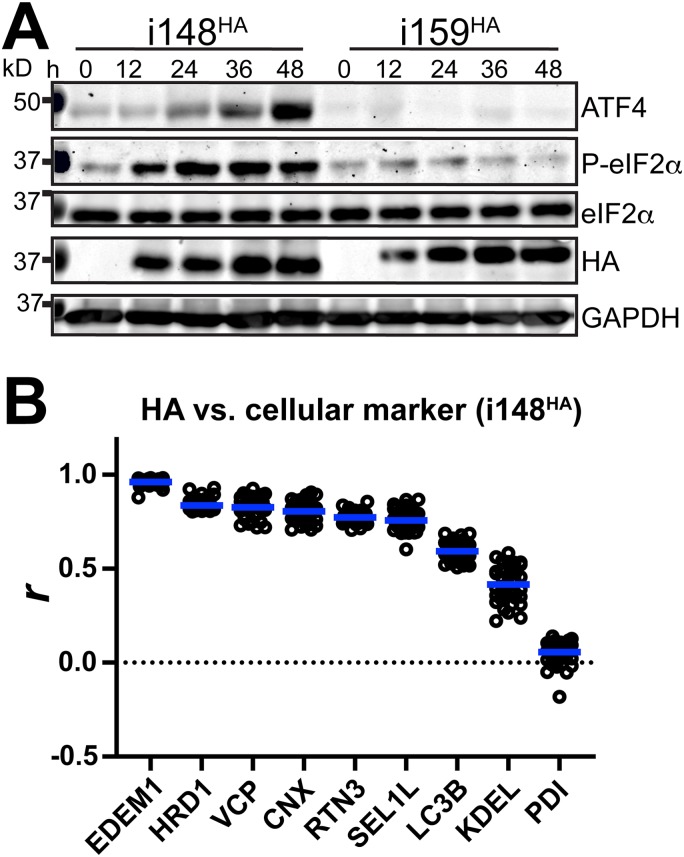
ISR activation accompanies redistribution of ER markers during ectopic expression of UL148. (A) Lysates of tet-on ARPE-19 cells expressing either UL148 or Rh159 fused to an HA tag, i148^HA^ or i159^HA^, respectively, were collected at the indicated times after doxycycline (dox) induction and analyzed by Western blotting for the expression of the indicated proteins and for the abundance of eIF2α phosphorylated at Ser51 (eIF2α-P) using a phospho-specific antibody. (B) Pearson’s correlation coefficient (*r*) values were calculated using NIH ImageJ software to estimate the degrees of colocalization between UL148 (HA signal) and the indicated cellular markers. A minimum of 30 cells were analyzed per marker. Arithmetic means for each colocalization analysis result are shown as blue lines, and data points for individual cells analyzed are plotted as circles.

In cells expressing the UL148 homolog Rh159 (i159^HA^), we detected uniform cytoplasmic distribution of ER markers, as well as of HA-tagged Rh159, similar to what we observed during infection with the recombinant HCMV TB_159^HA^ (Fig. 3). Furthermore, in this setting, the staining patterns for the ATG8 family proteins LC3B and GABARAP failed to indicate any notable structures. Consistent with our previous study showing that UL148, but not Rh159, is sufficient to activate the UPR (10), we observed accumulation of ATF4 and phosphorylated eIF2α following doxycycline (dox)-induced expression of UL148 but not Rh159 (Fig. 9A). Importantly, the intensity of HA signals detecting UL148 and Rh159 indicated that the two proteins accumulate at roughly comparable levels following dox induction. This argues against the possibility that differences in protein expression levels account for the differing effects on UPR activation and on the staining patterns for ER markers.

Pearson’s correlation coefficient values were calculated to quantify the extent of colocalization between UL148 and various cellular markers: EDEM1, HRD1, VCP, calnexin (CNX), RTN3, SEL1L, LC3B, and PDI (Fig. 9B). The results quantitatively buttress the confocal immunofluorescence results (Fig. S3). Based on these findings, we conclude that UL148 is sufficient to cause large-scale reorganization of ERQC markers. Moreover, our results imply that reorganization of ER markers into discrete structures may be related to the propensity of UL148 to cause ER stress.

To determine whether UL148-dependent ER remodeling could be visualized in real time using live cell imaging, we constructed lentiviral vectors that inducibly express either UL148 or Rh159 fused at their predicted C-terminal cytoplasmic tails to the enhanced green fluorescent protein (GFP) from Aequorea victoria (33). Following lentiviral transduction, we isolated puromycin-resistant ARPE-19 and used fluorescence-activated cell sorting to enrich for cells that expressed the GFP signal following dox treatment. The resulting cell populations, which inducibly expressed either UL148-GFP or Rh159-GFP, were designated i148^GFP^ and i159^GFP^, respectively.

In live cell imaging studies, we observed that the GFP signal in dox-induced i148^GFP^ cells first appeared in a reticular largely uniform pattern, which was readily visible at 5 h postinduction. However, by 9 h postinduction, punctate signals began to appear. These puncta were observed to traffic to sites of large-scale accumulation, where large fluorescent structures progressively increased in size up until the termination of the experiment at 19 h (see Fig. S4A; Movie S1). In contrast, i159^GFP^ cells exhibited a largely diffuse reticular GFP signal at all time points monitored subsequent to transgene induction (Fig. S4A; Movie S2). The live cell imaging results thus recapitulated the differences in the HA-staining patterns observed between dox-induced i148^HA^ and i159^HA^ cells following fixation, as well as those between HCMV expressing HA-tagged UL148 versus *UL148*-null comparator viruses (Fig. 1 and 3; Fig. S1 and S3).

10.1128/mBio.02110-19.4 FIG S4 Live cell imaging and analyses of UL148-GFP and Rh159-GFP following induced ectopic expression. (A) Live cell imaging. Tet-on ARPE-19 epithelial cells that inducibly express either UL148 or Rh159 fused to green florescent protein (GFP), i148^GFP^ and i159^GFP^, respectively, were induced for transgene expression using 100 ng/ml doxycycline (dox) and imaged using live-cell microscopy. Images from the selected time points (h posttreatment with dox [hpt]) are shown. Scale bars, 50 μm or 10 μm (for inset panels at upper left of each image, which are magnified 2.4× relative to the main image). See also Movies S2 and S^3^. (B) FRAP. i148^GFP^ and i159^GFP^ were dox induced for 24 h. Separate regions of GFP signal in selected cells were either photobleached (405-nm laser) or left unbleached, while a third region lacking GFP signal was chosen as a background reference and measured before and during fluorescence recovery after photobleaching (FRAP). Note that background signal was not plotted, because values were not resolvable from the *x* axis. GFP signal intensity is plotted over a time period (seconds) starting with an exposure at *t* = 0 (immediately before photobleaching) and including measurements taken every 2 s after photobleaching (0 to 20 s) until termination of the measurement series at t = 322 s (300 s of FRAP). Image series from the selected time points (seconds) immediately before and after bleaching, and during fluorescence recovery period are shown for each of the three analysis conditions. (C) UL148-GFP is stabilized by inhibition of proteasomal but not lysosomal degradation. Tet-on ARPE-19 cells that inducibly express UL148-GFP (i148^GFP^) were induced for 24 h by the addition of 100 ng/ml dox, after which the dox was washed out and medium containing either the proteasome inhibitor epoxomicin (20 μM) or the proton pump inhibitor folimycin (115 nM) was added; samples were harvested for Western blot analysis at the indicated times posttreatment (h posttreatment [hpt]) with folimycin, epoxomicin, or DMSO alone as a carrier control (1% or 0.1% to control for epoxomicin or folimycin treatments, respectively). no dox, a control sample in which UL148-GFP transgene was not induced. (D) Solubility analysis. Inhibitors of proteasomal or lysosomal degradation were applied as described for panel C except i148^HA^ cells were used instead of i148^GFP^ cells, epoxomicin was applied at 10 μM (from a 10 mM stock) instead of 20 μM final (from a 2 mM stock), DMSO was used as a vehicle control at 0.1% (vol/vol final) for comparisons against both folimycin and epoxomicin, and samples were lysed in RIPA buffer and subjected to high-speed centrifugation to separate detergent-insoluble (pellet) from -soluble (supernatant) fractions. no dox, supernatant fraction of lysate from uninduced control condition. (E) Pharmacological inhibition of VCP (p97) stabilizes UL148. VCP inhibitors ML241 (2 μM) and CB-5083 (1 μM) were compared against vehicle alone control (0.01% DMSO) for the potential to stabilize UL148^HA^ following washout of inducing agent. i148^HA^ cells were dox induced for 24 h, after which dox was washed out; then, medium lacking dox but containing the indicated VCP inhibitor was added. Lysates were collected at the indicated time points (h posttreatment [hpt]) following addition of VCP inhibitor. no dox, uninduced control lysate, lacking any VCP inhibitor treatment. Download FIG S4, TIF file, 2.5 MB. Copyright © 2019 Zhang et al. 2019 Zhang et al. This content is distributed under the terms of the Creative Commons Attribution 4.0 International license.

10.1128/mBio.02110-19.5 MOVIE S1 i148^GFP^ cells from 2 to 19 h post-dox induction. Download Movie S1, MPG file, 19.1 MB. Copyright © 2019 Zhang et al. 2019 Zhang et al. This content is distributed under the terms of the Creative Commons Attribution 4.0 International license.

10.1128/mBio.02110-19.6 MOVIE S2 i159^GFP^ cells from 2 to 19 h post-dox induction. Download Movie S2, MPG file, 10.6 MB. Copyright © 2019 Zhang et al. 2019 Zhang et al. This content is distributed under the terms of the Creative Commons Attribution 4.0 International license.

### UL148-GFP structures exhibit poor recovery of fluorescence after photobleaching.

To determine whether the protein contents of the UL148-induced structures exhibit decreased mobility compared to that in nonperturbed ER regions, we carried out fluorescence recovery after photobleaching (FRAP) studies. We photobleached regions of GFP signal in i148^GFP^ or i159^GFP^ ARPE-19 cells that had been induced for transgene expression for 24 h and then monitored recovery of fluorescence over a 5-min time period. In Rh159-GFP-expressing cells (i159^GFP^), fluorescence nearly recovered to prebleach levels within 3 min and by 5 min, was fully recovered (Fig. S4B). In contrast, when we photobleached a prominent UL148-GFP structure, the GFP signal failed to appreciably recover fluorescence during the same 5-min time period. Nonetheless, regions of reticular GFP signal from an i148^GFP^ cell, which presumably represent ER regions not involved in an anomalous structure, recovered fluorescent signal with kinetics similar to those observed during Rh159-GFP expression. These results indicate that UL148-GFP within the structures cannot rapidly be exchanged with UL148-GFP from other portions of the organelle, which suggests that the induced UL148 structures do not exchange their contents as efficiently as unperturbed ER.

### Ectopically expressed UL148 is primarily degraded by proteasomes.

The UL148 ER structures were found to be enriched with proteins such as HRD1, SEL1L, EDEM1, and VCP (Fig. 3, 4, and 9B; Fig. S3), which are posited to play key roles in ERAD, a process during which misfolded glycoproteins are recognized, processed, and dislocated across the ER membrane for degradation at cytosolic proteasomes. However, we also detected elements of the machinery for autophagy in close association with the UL148 structures. Namely, the mammalian ATG8 ortholog GABARAP colocalized with UL148 during infection (Fig. 3 and 4), and both GABARAP and another ATG8 ortholog, LC3B, colocalized with UL148 at the ER structures during ectopic expression of UL148 (Fig. 9B; Fig. S3). Therefore, we wished to determine whether UL148 is degraded by the proteasome, as would be consistent with conventional ERAD, or by the lysosome, which would suggest a role for autophagy-related processes, such as selective autophagy of the ER (34), in dispensing with UL148 and, presumably, in resolving the ER perturbations.

As a first step, we conducted a live cell imaging “washout” experiment in which i148^GFP^ cells were induced for transgene expression for 24 h, after which the growth medium containing the transgene-inducing agent (dox) was replaced with medium lacking dox. During a 21-h imaging period following dox washout, we observed the structures to become progressively smaller as the reticular and punctate GFP signals gradually abated (Fig. S4C). Because these results suggested that UL148-GFP structures could be resolved over time, we next induced UL148-GFP for 24 h, washed out the inducing agent (dox), and then applied either epoxomicin (20 μM), an irreversible inhibitor of the proteasome (35), or folimycin (115 nM), a proton pump inhibitor that blocks autophagosome maturation and impedes lysosome-dependent degradation (36, 37). Of the two inhibitors, only epoxomicin stabilized UL148 (Fig. S4C). Although folimycin treatment had no obvious effect on UL148-GFP levels, the drug markedly increased the levels of LC3B-II, as would be expected with the lysosomal proton pump inhibitor. When folimycin and epoxomicin treatments were compared in similar dox washout experiments in which detergent soluble and insoluble fractions of UL148 were analyzed separately, epoxomicin showed its most pronounced effect on the detergent-soluble fraction of UL148 (Fig. S4D).

To address whether UL148 is being targeted to the proteasome via the ERAD pathway, we asked whether inhibition of the AAA^+^ ATPase VCP (p97), would phenocopy the stabilization seen with epoxomicin. VCP plays a crucial role in the dislocation of ERAD substrates across ER membranes (38), and its inhibition impedes ERAD (reviewed in reference 39). For these studies, we turned to two chemically distinct VCP inhibitors, CB-5083 (40, 41) and ML241 (42), both of which block ERAD without detectably impairing autophagy (39, 40, 42); in fact, CB-5083 is thought to enhance autophagy (40). We induced the expression of HA-tagged UL148 from i148^HA^ cells for 24 h and subsequently washed out the inducing agent (dox). The medium was replaced with medium containing CB-5083 (1 μM), ML241 (2 μM), or dimethyl sulfoxide (DMSO) vehicle alone (0.01%). Each of the VCP inhibitors markedly stabilized UL148, sustaining detectable levels of HA signal out to 24 h post-dox washout, while UL148 became virtually undetectable in the DMSO control-treated cells by 18 h postwashout. From these results, we conclude that ectopically expressed UL148 is primarily degraded by a proteasome-dependent pathway, likely via VCP-dependent ERAD.

### UL148 requires the integrated stress response to cause ER reorganization.

We previously reported that UL148 triggers the UPR during ectopic expression and that UL148 contributes to activation of the PKR-like ER kinase (PERK) and inositol requiring enzyme 1 (IRE1) during infection (10). The literature suggests that formation of ERQC compartments requires PERK (43). PERK responds to ER stress by activating the integrated stress response (ISR). In particular, PERK phosphorylates eIF2α at Ser51, and the accumulation of phosphorylated eIF2α (eIF2α-P) globally attenuates mRNA translation while stimulating the translation of a subset of mRNAs, such as those encoding ATF4 and CHOP, which play roles in cellular adaptation to stress (44). Stress-regulated translation of such mRNAs involves small upstream open reading frames (uORFs) in their 5′ untranslated regions that suppress translation under basal conditions (45, 46).

**FIG 10:**
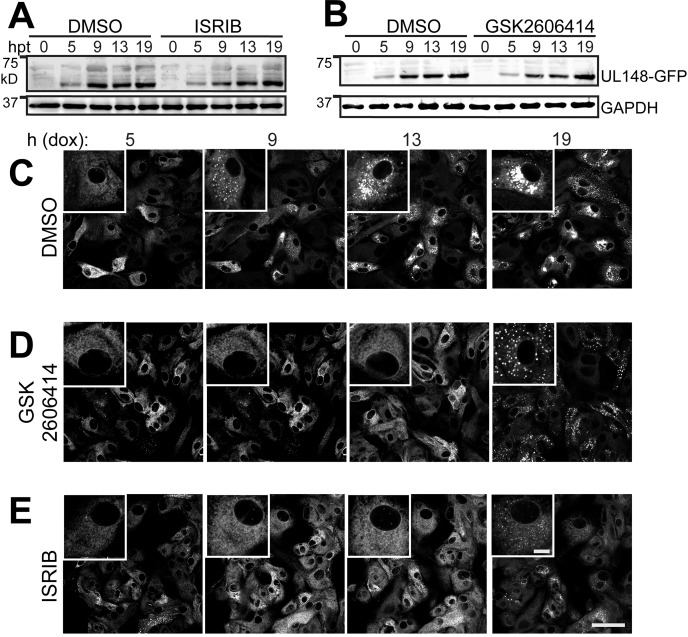
Inhibition of the integrated stress response impedes UL148-mediated ER remodeling. (A and B) Tet-on ARPE-19 epithelial cells that inducibly express UL148 fused to green florescent protein (GFP), i148^GFP^, were induced for transgene expression using 100 ng/ml doxycycline (dox) in the presence of DMSO carrier, ISRIB (200 nM), or GSK2606414 (1.1 μM) and monitored by anti-UL148 Western blotting for expression of UL148-GFP over a series of time points (h posttreatment with dox [hpt]). (C to E) Live cell imaging of UL148-GFP expression patterns in the presence of ISRIB (200 nM), GSK2606414 (1.1 μM), or DMSO vehicle (0.01%). Scale bars, 50 μm or 10 μm (for inset panels at upper left of each image, which are magnified 2.4× relative to the main image). See also Movies S3 to S^5^ in the supplemental material.

To examine whether ER remodeling in response to UL148 requires the ISR, we turned to a well-characterized small molecule inhibitor of stress-regulated translation, ISRIB (47–51). ISRIB is thought to act as a “molecular staple” that holds the guanine nucleotide exchange factor eIF2B in an active decameric configuration (49–52), such that eIF2B will continue to generate the ternary complex (eIF2·GTP·Met-tRNAi) necessary for new cycles of translational initiation, despite the presence of eIF2α-P. Because PERK is the kinase that phosphorylates eIF2α in response to ER stress (53, 54), we also tested for effects of the PERK inhibitor GSK2606414 (55). Having confirmed that ISRIB and GSK2606414 do not negatively impact UL148-GFP expression following dox induction (Fig. 10), we treated i148^GFP^ cells with 200 nM ISRIB, 1.1 μM GSK2606414, or DMSO vehicle control and carried out live cell imaging following dox induction.

10.1128/mBio.02110-19.7 MOVIE S3 i148^GFP^ cells, 0.01% DMSO, 2 to 19 h post-dox induction. Download Movie S3, MPG file, 16.5 MB. Copyright © 2019 Zhang et al. 2019 Zhang et al. This content is distributed under the terms of the Creative Commons Attribution 4.0 International license.

ISRIB and GSK2606414 virtually abolished the formation of UL148 puncta through 13 h postinduction (dox addition), a time point at which the DMSO control condition showed abundant large structures (Fig. 10C to E; Movies S3 to S^5^). Based on our findings (Fig. 1, 3, 5, 7, and 9B; Fig. S3), we interpret the formation and subsequent large-scale aggregation of UL148-GFP puncta to faithfully indicate ER reorganization in response to UL148. Therefore, these results suggest that pharmacological inhibition of either the ISR or PERK prevent UL148 from reorganizing the ER, which implies that ER remodeling in response to ER stress requires stress-regulated translation.

We next asked whether these pharmacological agents would prevent UL148-dependent ER remodeling in the physiologically authentic context of HCMV infection. Our live cell imaging studies from ISRIB and GSK2606414 treatments indicated that, despite inhibitor treatment, UL148-GFP puncta began to appear by ∼19 h posttreatment (Fig. 10; Movies S4 and S^5^), which suggested that the pharmacological activity of the drugs might diminish over time. To ensure that blockade of PERK and the ISR would be sustained over the 4-day period it takes for structures to fully form during HCMV infection, we replenished drug treatments every 24 h.

10.1128/mBio.02110-19.8 MOVIE S4 i148^GFP^ cells, 1.1 μM GSK2606414, 2 to 19 h post-dox induction. Download Movie S4, MPG file, 16.8 MB. Copyright © 2019 Zhang et al. 2019 Zhang et al. This content is distributed under the terms of the Creative Commons Attribution 4.0 International license.

**FIG 11:**
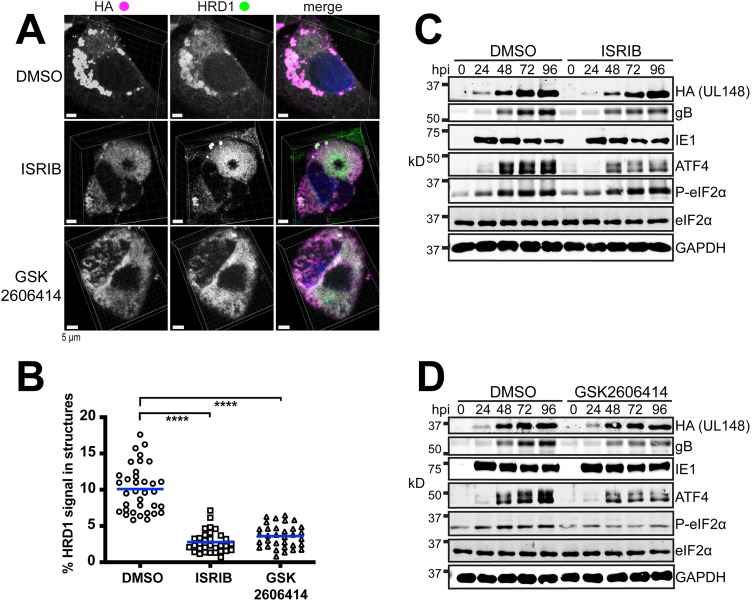
Inhibition of the ISR prevents the coalescence of HRD1 and UL148 into discrete structures during infection. (A) Representative 3D maximum intensity projections of confocal imaging Z-stacks obtained from cells infected at an MOI of 1 for 96 h with HCMV strain TB40/E carrying an HA-tagged *UL148*, TB_148^HA^, and maintained in the presence of ISRIB (200 nM), GSK2606414 (1.1 μM), or DMSO carrier alone (0.1% [vol/vol]). In merged images, HA signal is shown in magenta, HRD1 in green, and DAPI counterstaining in blue. (B) The percentages of HRD1 antibody signals involved in discrete structures at 96 hpi were calculated for a minimum of 30 cells per condition using Imaris x64 9.3.0 software. Statistical significance was determined using a one-way ANOVA followed by Tukey’s posttest; ****, *P* < 0.0001. The arithmetic mean for colocalization analysis results are shown as blue lines with data points for individual cells analyzed plotted as circles, squares, or triangles, as indicated. (C and D) Western blot analyses of fibroblasts infected at an MOI of 1 with TB_148^HA^ and maintained in the presence of ISRIB (200 nM), GSK2606414 (1.1 μM), or DMSO carrier alone (0.01%); hpi, h postinfection. Note that a phospho-specific antibody was used for detection of eIF2α phosphorylated at Ser51 (eIF2α-P).

At 4 dpi, ER structures were readily visible under the DMSO control treatment condition but virtually abolished under the ISRIB and GSK2606414 conditions (Fig. 11A). To quantify the effects, we obtained Z-stacks from a minimum of 30 cells per treatment condition and used Imaris 3D image analysis software to calculate the percentage of HRD1 signal that coalesced into discrete structures during infection. The results indicated highly significant differences between the DMSO vehicle control setting and treatment with either GSK2606414 or ISRIB (Fig. 11B). Under the DMSO control condition, on average, 10% of the HRD1 signal was found to be associated with the UL148 structures (arithmetic mean; range, 5.8% to 17.6%), as indicated by structures delimited by HA signal detecting UL148. In the presence of ISRIB or GSK2606414, however, these values were reduced to 2.8% (range, 0.69% to 7.1%) and 3.6% (range, 0.8% to 6.5%), respectively. Reassuringly, roughly equivalent levels of UL148 were detected in Western blot analyses of protein extracts from drug-treated and DMSO control conditions. Therefore, differences in UL148 expression are unlikely to explain the observed failure of UL148 and HRD1 to coalesce into ER structures during inhibition of either the ISR or PERK. Meanwhile, ISRIB and GSK2606414 treatments both led to reduced levels of ATF4 accumulation, while the PERK inhibitor alone was able to reduce the levels eIF2α-P, as indicated by a phospho-specific antibody. Therefore, each of the drugs caused the expected effects on the PERK, eIF2α-P, and ATF4 axis (Fig. 11C and D).

A modest reduction in the levels of the viral envelope glycoprotein B (gB) was observed during treatments with ISRIB or GSK2606414. This may suggest that the ISR is required for optimal expression of certain viral envelope glycoproteins. Even though UL148 promotes ATF4 expression, likely via PERK activation (10), viruses that lack *UL148* nonetheless still cause high levels of ATF4 accumulation at late times during infection, which is when viral late gene products, such a gB, are robustly expressed (10). Hence, the activity of PERK and/or of the ISR may be required for optimal expression of viral envelope glycoproteins. Regardless, the PERK inhibitor and ISRIB each prevented the appearance of discrete UL148 ER structures while failing to substantially affect UL148 expression. Therefore, the effects of the two inhibitors on UL148-dependent ER reorganization were consistent with those seen during ectopic expression of the protein. Taken together, our findings argue that the ISR is required for UL148 to cause remodeling of the organelle.

## DISCUSSION

The ER structures that we have identified are noteworthy in several regards. First, this example of virally induced ER remodeling is wholly dependent on a single viral gene product, UL148. Of course, there are other examples of individual viral gene products that are necessary to grossly perturb ER morphology during infection which are also sufficient to induce such perturbations (e.g., see references 30, 31, 56, and 57, reviewed in reference 32). For instance, expression of the envelope protein J13Lp from African swine fever virus is sufficient to cause collapse of ER cisternae in a manner that depends on disulfide bonds between antiparallel arrays of J13Lp N termini, which assemble across the ER lumen (31). Hepatitis C virus (HCV) causes the formation of a rough ER-derived “membranous web” consisting of clusters of vesicles embedded within a membrane-rich matrix and is thought to provide a hub for the assembly of viral RNA replication complexes (58, 59). The HCV protein NS4B is sufficient to induce these structures (58), or at least to drive the formation of vesicles (60).

Second, the ER structures induced by UL148 are, to the best of our knowledge, a hitherto undescribed ultrastructural characteristic of the HCMV-infected cell, which is surprising given their size, scale, and prominence. The average volume of the structures at 96 hpi amounts to roughly 60% of the size of the nucleus of an uninfected human fibroblast (∼500 μm^3^ [61]). Third, although UL148 expression is accompanied by UPR activation, ER reorganization occurs without killing the host cell, even in the setting of ectopic expression when viral functions that inhibit programmed cell death are absent. Moreover, UL148 does not affect the yield of infectious virus during replication in fibroblasts, since *UL148*-null mutant viruses replicate indistinguishably from wild-type parental virus in this cell type (1, 8). As with all enveloped viruses, HCMV requires the host cell secretory pathway to fold and process enormous quantities of viral envelope glycoproteins that endow progeny virions with the molecular machinery for infectivity. It is intriguing to consider how the infected fibroblast tolerates spatial reorganization of ER membranes and of the associated glycoprotein quality control machinery without impacting the production of infectious progeny virions.

Finally, certain aspects of ER reorganization invoked by UL148 may be novel to cell biology. In particular, the densely packed collapsed ER in close association with varicosities that we observe to depend on UL148 during infection appear to differ from the ER structures induced by other ER perturbagens. For instance, a hereditary form of childhood cirrhosis caused by the *Z* allele of alpha 1 antitrypsin (*SERPINA*) is characterized by polymerization of the mutant gene product (Z-A1AT) within the ER (27), leading to its accumulation in membrane-delimited inclusions (27, 28, 62). However, Z-A1AT inclusions are not observed to associate with regions of collapsed ER, and Z-A1AT does not suffice to activate the UPR, even though its expression sensitizes cells to other triggers of ER stress (28, 63).

HCMV encodes at least one other ER-resident immunevasin that activates the UPR, US11 (64). UL148 binds the NK cell and T-cell costimulatory ligand CD58 to prevent its transport to the cell surface, while US11 targets the heavy chain of class I major histocompatibility complex (MHC) for ER-associated degradation (65, 66). UL148 causes CD58 to accumulate as an immature glycoform, presumably within the ER, but does not lead to any obvious decrease in its overall abundance (9). Although intracellular forms of CD58 are, in our hands, refractory to detection by standard indirect immunofluorescence protocols (not shown), it seems reasonable to hypothesize that UL148 sequesters CD58 into the unusual ER structures that it induces. Nonetheless, recent work from our laboratory identified charged-cluster-to-alanine mutants of UL148 that prevent cell surface presentation of CD58 but fail to reorganize the ER (67). Likewise, the chimpanzee CMV homolog of UL148 appears to prevent cell surface presentation of CD58 without reorganizing the ER (67). Although we cannot exclude the possibility that ER reorganization may prove crucial for a hitherto unidentified immune evasion function of UL148, these observations argue that ER remodeling is not required for CD58 retention, *per se*.

Although the capacity of UL148 to retain CD58 may be functionally separable from its peculiar effects on the morphology and organization of the ER, our findings may suggest a mechanism to explain the influence of UL148 on the tropism of the virus for epithelial cells. These effects, exemplified by an ∼100-fold replication advantage of *UL148*-null viruses during infection of epithelial cells, correlate with decreased expression of glycoprotein O (gO), a viral envelope glycoprotein, both in virions and in infected cells (1). We previously reported that gO behaves as a constitutive ERAD substrate during infection and that immature newly synthesized forms of gO show enhanced stability in the presence of UL148 (8). Our findings herein show that UL148 causes large-scale sequestration of cellular factors important for ERAD, such as the ER mannosidase EDEM1 and the E3 ubiquitin ligase SEL1L/HRD1, into large membranous structures. Moreover, the reticular ER, as indicated by the staining patterns for calreticulin, PDI, and the KDEL motif, appears to remain largely intact following UL148-induced ER reorganization (Fig. 3; see also Fig. S3 in the supplemental material). Since these observations indicate that UL148 depletes ERAD factors from the ER during the formation of the unusual structures, regions of ER not drawn into the structures might be expected to offer a more permissive folding environment for polypeptides, such as gO, which either fold slowly or inefficiently assemble into multiprotein complexes.

Based on these observations, one might hypothesize that UL148 alters ER proteostasis by physically dislocating, or sequestering, key elements of the “mannose removal time clock” system that marks poorly folding glycoproteins for destruction via ERAD (68, 69). Because gO is both the defining subunit of the heterotrimeric gH/gL/gO envelope glycoprotein complex that governs HCMV entry and cell tropism (5–7, 70, 71) and a constitutive ERAD substrate (1, 8), ER reorganization might be required for the effects of UL148 on HCMV cell tropism. Going forward, it will be crucial to determine whether classical ERAD substrates, such as the null Hong Kong variant of alpha 1 antitrypsin (72, 73) or ribophorin 332 (74, 75), are stabilized during UL148 expression, as would be predicted if UL148 shifts ER proteostasis to negatively modulate ERAD.

In addition to having found that factors involved in proteasomal ERAD, such as HRD1 and EDEM1, are enriched at the UL148 structures, we observed that GABARAP, a mammalian ortholog of yeast ATG8, localizes to the UL148 ER structures during infection and that GABARAP and another ATG8 ortholog, LC3B, both associate with the structures during ectopic expression of UL148. Although we have yet to determine whether lipidated forms of GABARAP or LC3B (e.g., LC3-II) associate with the ER structures, their presence may suggest roles for autophagy-related pathways in UL148-dependent reorganization of the organelle. Since our results indicate that UL148 is most likely degraded by a proteasome and VCP-dependent pathway and not by lysosomes (Fig. S4C to E), it seems unlikely that selective autophagy of the ER is directly involved in recycling or degrading these ER structures. Nonetheless, ATG8 family proteins may contribute to the formation of the globular ER structures, since we observed trafficking of UL148-GFP puncta to form large aggregates during live cell imaging (Movies S1 and S3). The literature indicates that misfolded proteins traffic in a microtubule (MT)-dependent manner to form pericentriolar structures termed aggresomes, which, in the case of ERAD substrates such as the ΔF508 mutant of the cystic fibrosis transmembrane conductance regulator (CFTR), contain deglycosylated protein that presumably has already undergone dislocation from the ER (76, 77). ATG8 family proteins not only play roles in the degradation of substrates via macroautophagy, but also bind MTs and recruit machinery for MT-dependent transport of cargos (78–80). Therefore, GABARAP and LC3B may be important for the recruitment of cellular machinery that transports perturbed ER cargoes to sites of large-scale accumulation.

Although our results argue that the ISR is required for ER reorganization during UL148 expression, precisely how UL148 triggers ER stress remains unknown. UL148 has been found to copurify from cells with SEL1L, a component of the ERAD machinery. Thus, it seems plausible that UL148 may inhibit the HRD1/SEL1L complex, which would cause the buildup of unfolded proteins and thus trigger the UPR. However, inhibition of ERAD in and of itself seems unlikely to account for the formation of ER structures. Another nonmutually exclusive possibility is that UL148 multimerizes or aggregates in a manner that constricts the ER lumen. For instance, the assembly of UL148 molecules on opposite sides of the ER lumen might constrict the organelle in a manner consistent with the collapsed regions of ER observed in our EM results (Fig. 5 and 7).

Additional work will be needed to decipher the molecular mechanisms by which UL148 causes reorganization of the ER and to determine its biological relevance in the context of viral infection. Nonetheless, our collective data indicate that UL148, when fused to a fluorescent protein (FP), suffices both to trigger and to indicate the presence of a functional ISR (Fig. 9 and 10; Movies S3 to S5). Hence, UL148-FP fusions may prove useful in high-throughput chemical-genetic screens to identify novel small molecule inhibitors of the ISR as well as to identify cellular genes involved in stress-dependent remodeling of the ER. Moreover, given the broad importance macromolecular aggregation under pathological conditions such as neurodegenerative diseases (81), certain of which also involve defects in ER proteostasis and aberrant activation of the UPR, UL148 may hold promise as tool to discover new agents to ameliorate disease.

Notably, UL148 is among the subset of viral genes dispensable for replication in cultured cells that are never (82) or almost never (83) spontaneously disrupted in clinical HCMV genomes recovered from clinical samples. This suggests that UL148 plays essential roles during natural infection. Given that ER reorganization appears to be dispensable for CD58 retention (67), it would not be unreasonable to hypothesize that the striking effects of UL148 on the organelle may reflect additional roles of UL148 that contribute to viral persistence *in vivo*.

## MATERIALS AND METHODS

### Cells and virus.

Human telomerase reverse transcriptase (hTERT)-immortalized human foreskin fibroblasts (8), derived from ATCC HFF-1 cells (SCRC-1041), were maintained in Dulbecco’s modified Eagle’s medium supplemented with 5% to 10% newborn calf serum (Millipore Sigma) and antibiotics (complete DMEM) exactly as described previously (8). i148^HA^ and i159^HA^ ARPE-19 epithelial cells (10), which upon treatment with 100 ng/ml doxycycline express HA-tagged UL148 and Rh159, respectively, were likewise maintained in complete DMEM. For live cell imaging studies, i148^HA^ and i159^HA^ ARPE-19 were maintained in Opti-MEM (Thermo Fisher) supplemented with 3% certified tet-approved fetal bovine serum (FBS), 20 μg/ml gentamicin sulfate, 1 μg/ml puromycin HCl, and 10 μg/ml ciprofloxacin HCl. Telomerase-immortalized rhesus fibroblasts (84) were a kind gift from Peter A. Barry and were maintained in complete DMEM. The acute monocytic leukemia cell line THP-1 (TIB-202) was obtained from ATCC (Manassas, VA) and maintained as suspension cultures in RPMI 1640 medium supplemented with 10% FBS (Millipore Sigma) and antibiotics (10 μg/ml ciprofloxacin and 25 μg/ml gentamicin). THP-1 cells were differentiated into adherent macrophages by incubating for 48 h in the presence of 100 nM 2-*O*-tetradecanoylphorbol 13-acetate (Millipore Sigma) and subsequently infected with the indicated viruses at an MOI of 5 50% tissue culture infective dose (TCID_50_)/cell.

Infectious bacterial artificial chromosome (BAC) clones of the following HCMV strains were used for this study: TB40/E (also known as TB40-BAC4 or TB_WT) (11) as well as its derivatives TB_148^HA^ and TB_148_STOP_, which were used in our previous studies (1, 8, 10); TR (TR*gfp*) (85, 86); Merlin repaired for *UL128* and *RL13* harboring *tetO* sequences upstream of *UL131* (pAL1393) (87); AD169 repaired for *UL131* (AD_r131) (1, 88); and AD_r131_148^HA^, a derivative of AD_r131 that carries a full-length UL148 (from strain TB40/E) tagged with an HA epitope at the original *UL148* locus (8). An infectious BAC clone of rhesus CMV (RhCMV) strain 68-1 (89) and a derivative that expresses an HA-tagged Rh159 (details below) were also used for certain experiments.

The methods used for reconstitution of HCMV from purified BAC DNA, cultivation of virus, and preparation of stocks, including ultracentrifugation through sorbitol cushions and determination of infectious titers by tissue culture infectious dose 50 (TCID_50_) assay have been described elsewhere (8, 10).

### Construction of recombinant viruses and new plasmids for the study.

New recombinant viruses for this study were derived from BAC-cloned HCMV and RhCMVs using *en passant* mutagenesis in GS1783 Escherichia coli (90, 91). Recombinant BACs were confirmed by Sanger DNA sequencing of the modified regions, which was performed by Genewiz, Inc. (Piscataway, NJ) (not shown). Oligonucleotide primers for construction of recombinant viruses and plasmids were custom synthesized by Integrated DNA Technologies (Coralville, IA); sequences are provided in Table S1A in the supplemental material. Type II restriction endonucleases, T4 DNA ligase, and Gibson assembly reagents (NEB HiFi Assembly Master Mix) for generation of recombinant DNAs and for routine molecular cloning and subcloning procedures were obtained from New England BioLabs (Ipswich, MA). KOD Hot Start DNA polymerase (EMD Millipore) was used for all PCRs.

10.1128/mBio.02110-19.10 TABLE S1 (A) Oligonucleotides and synthetic DNAs. (B) Antibodies used in immunofluorescence microscopy. Download Table S1, DOCX file, 0.1 MB. Copyright © 2019 Zhang et al. 2019 Zhang et al. This content is distributed under the terms of the Creative Commons Attribution 4.0 International license.

To construct TB_159^HA^, a strain TB40/E derivative in which the UL148 open reading frame is replaced by Rh159, we carried out the following steps. Primers Rh159_Fw and Rh159_HA_Rv were used to amplify and HA tag the *Rh159* ORF from pcDNA3.1+_Rh159_IRES_GFP (a gift from Klaus Früh, OHSU). The PCR product was inserted into EcoRV-digested pEF1α V5 His C (Invitrogen) by Gibson assembly. An ISce-I excisable kanamycin resistance cassette was amplified from the TB_148^HA^_*ISce-Kan* integrate BAC (1) using primers PpuISceKanGibs_Fw and PpuISceKanGibs_Rv and Gibson assembled into PpuMI-digested pEF1α_Rh159 plasmid to yield plasmid pEF1α_Rh159_ISceKan. Primers Rh159_Fw_recomb and Rh159_Rv_recomb were used to generate a PCR product from template plasmid pEF1α_Rh159_ISceKan. The PCR product was electroporated into GS1783 E. coli harboring the TB_148^HA^ BAC, and kanamycin-resistant “integrate” colonies were isolated on Luria-Bertani agar plates. The integrates were subsequently resolved to “scarlessly” remove the kanamycin resistance marker by standard *en passant* protocols (90, 91), yielding TB_Δ148_Rh159^HA^, which we abbreviate herein as TB_159^HA^. TB_159^HA^ was sequence confirmed using primers TB_159HAseq_F and TB_159HAseq_R. Similar strategies were used to modify the RhCMV 68-1 BAC (89) to incorporate sequences encoding an HA epitope immediately before the stop codon of *Rh159*. An *en passant* strategy in which a shuttle plasmid carrying I-SceI-Kan^r^ disrupted by in-frame nonsense codons was used to insert premature nonsense codons in the *UL148* coding DNA sequence (CDS) in the context of HCMV strains Merlin (pAL1393) and TR (TR*gfp*) and was applied as described previously for generating TB_148_STOP_ (8).

To construct tet-on lentivirus vector plasmids containing UL148 fused to GFP, primers Gibs_eGFP_Rv and 148_eGFP_Fw were used to amplify the *egfp* gene from a double-stranded DNA (dsDNA) gBlock, EGFP-P2A-3XHA, synthesized by Integrated DNA Technologies (Coralville, IA), a gift from Matthew D. Woolard (LSU Health Sciences Center, Shreveport, LA). In a separate PCR, the *UL148* gene was amplified from plasmid pcDNA3.1-UL148^HA^ (10) using primers Gib_148_Fw and 148_noStopRv. The two products were assembled together with EcoRV-opened pcDNA3.1(+) (Invitrogen) to produce pcDNA3.1-UL148-GFP. After confirming the absence of spurious mutations by Sanger DNA sequencing (Genewiz) (not shown), the *UL148-gfp* cassette from pcDNA3.1-UL148-GFP was released by EcoRI and NotI digestion and ligated into pOUPc turboRFP (tRFP)-link plasmid, a derivative of pOUPc (10) that was modified to add EcoRI and NotI sites.

To express Rh159 fused to eGFP, primers Gibs_eGFP_Rv and 159_eGFP_Fw were used to amplify the GFP gene from EGFP-P2A-3XHA, and in a separate PCR, primers Gibs_159_Fw and 159_noStop_Rv Rh159 were used to amplify *Rh159* from pcDNA3.1-Rh159^HA^ (10). The latter two PCR products were assembled together with EcoRV-linearized pcDNA3.1(+) using Gibson assembly, resulting in plasmid pcDNA3.1-Rh159. The latter plasmid was used as the template in a PCR with primers Gibs_Age_159_Fw and Gibs_Mlu_GFP_Rv. The resulting PCR product was Gibson assembled into pOUPc (10) that had been linearized by MluI and AgeI.

### Drug treatments.

The PERK inhibitor, GSK2606414 (55), doxycycline hyclate, and folimycin were obtained from MilliporeSigma (Burlington, MA). ISRIB (47–51), ML241 HCl (ML241), and CB-5083 were obtained from APExBio (Boston, MA); epoxomicin was procured from Selleck Chemicals (Houston, TX). ISRIB was dissolved in DMSO to make a 10,000× stock solution (2 mM) and used at a final working concentration of 200 nM. GSK2606414 was dissolved in DMSO to make a 10,000× stock solution (11 mM) and used at a final working concentration of 1.1 μM. Folimycin was prepared as a 1,000× (115.46 μM) stock solution in DMSO and used at a final concentration of 115 nM. For the experiment shown in Fig. S4C, epoxomicin was prepared as a 2 mM (100×) stock solution in DMSO and applied to cells at a final concentration of 20 μM. For the experiment shown in Fig. S4D, a 10 mM (1,000×) stock solution was used, and the final concentration applied to cells was 10 μM. ML241 was prepared as a 20 mM (10,000×) stock solution in DMSO and was applied to cells at 2 μM. CB-5083 was prepared as a 10 mM (10,000×) stock solution and was applied to cells at 1 μM.

### Confocal microscopy and live cell imaging.

Confocal indirect immunofluorescence microscopy imaging on fixed cells was carried out using a Leica TCS SP5 spectral confocal microscope (Leica Microsystems, Heidelberg, Germany) using a Leica HCX PL APO CS 63×/1.4 to 0.6 numerical aperture (NA) lens objective under oil immersion, except for the image shown in Fig. 1C, which was captured on a Nikon SIM-E and A1R confocal microscopy system (Nikon Instruments, Melville, NY) using a Nikon SR Apo total interna-reflection fluorescence (TIRF) 100×/1.49 NA lens objective under oil immersion. Images for different fluorophore channels were acquired using sequential scanning. Direct immunofluorescence live cell imaging data were collected using the Nikon SIM-E microscope equipped with a Nikon Apo 60×/1.40 NA oil immersion objective. The three-dimensional (3D) projection shown in Fig. 1 was generated by NIS-Elements AR Analysis 4.60.00 (64-bit) software (Nikon) from Z-stacks captured on the Nikon SIM-E/A1R microscope using a Nikon SR Apo TIRF 100×/1.49 NA lens objective. For Fig. 11, 3D projections were generated by Imaris x64 9.3.0 software (Bitplane, Inc.) in maximum intensity projection (MIP) mode.

For fixed cell imaging experiments other than those shown in Fig. S1D, cells were seeded on 12-mm, circular, number-1 thickness microscope cover glass (200121; Azer Scientific, Morgantown, PA). At various times posttreatment and or postinfection, cells were washed with phosphate-buffered saline (PBS) (137 mM NaCl, 2.7 mM KCl, 10 mM Na_2_HPO_4_, and 1.8 mM KH_2_PO_4_, pH 7.4), fixed for 15 min at room temperature in PBS containing 4% (wt/vol) paraformaldehyde (Fisher Scientific, Waltham, MA), washed in PBS, permeabilized for 10 min using 0.1% Triton X-100 (in PBS), subsequently washed again in PBS, and then blocked for 45 min at 37°C in PBS containing 5% (vol/vol) normal goat serum (Rockland Immunochemicals, Limerick, PA). Cells were then washed three times in PBS followed by incubation in 1% human Fc block (BD Biosciences, San Jose, CA) in PBS for an additional 45 min at 37°C. Cells were then incubated in the presence of primary antibodies for 1 h at 37°C or 4°C overnight and then washed three times with PBS containing 0.1% Tween 20 (PBST) for 5 min per wash. Alexa Fluor-labeled goat polyclonal secondary antibodies (all from Thermo Fisher Invitrogen, Waltham, MA) (Table S1B) were used for secondary detection. The slides were then mounted using Prolong Gold anti-fade reagent containing DAPI (Thermo Fisher) and placed under a Leica TCS SP5 confocal microscope for image acquisition using a Leica 63× oil immersion lens objective (Leica Microsystems).

For indirect immunofluorescence staining results of primary clinical isolates (Fig. S1D), four clinical HCMV isolates obtained by routine testing of throat swabs from patients of the Ulm University Medical Center were provided by the diagnostic laboratory of the Institute of Virology in Ulm. Sample material was applied to human fibroblasts and incubated for several days until HCMV-positive cells were detected. Infected cells were then seeded together with uninfected fibroblasts, incubated for up to 5 days until plaques were formed, and processed for indirect immunofluorescence staining. ERQC compartments were detected by staining for calnexin (CNX) (E10, mouse, 1:50 dilution; Santa Cruz Biotechnology), the cVAC was detected by staining for HCMV pUL71 (rabbit, 1:500). The secondary antibody used for the detection of CNX was Alexa Fluor 555-labeled goat anti-mouse IgG (1:1,000), and for pUL71 detection, Alexa Fluor 488-conjugated goat anti-rabbit IgG (1:1,000) was used. Confocal images were acquired using the 63× lens objective of a Zeiss Observer Z1 fluorescence microscope equipped with Apotome and Zen software 2.3 (Carl Zeiss Microscopy GmbH, Jena, Germany).

### FRAP.

FRAP studies were carried out on ARPE-19 cells from the i148^GFP^ and i159^GFP^ populations as follows. Cells were seeded as described above for live cell imaging, induced for transgene expression using dox (100 ng) for 24 h, and then placed on an incubated sample stage of a Nikon A1R SIM-E imaging system equipped with an SR Apo TIRF 100×/1.49 NA lens objective (Nikon). Three rectangular regions in image fields were defined for measurement of (i) background signal, and of two regions with comparable initial GFP signals, (ii) one designated a control region (no photobleaching), and another for (iii) photobleaching and recovery of signal after photobleaching. Photobleaching of selected regions was carried out for 20 s using 405-nm laser light from a LU-N4 laser fiber (Nikon) (power at source, 15 mW; power at objective, 8 mW). Images and signal intensity measurements were captured every 2 s at a rate of 1 frame per s (488 nm excitation, fluorescein isothiocyanate [FITC] channel) starting immediately before photobleaching (*t* = 1), and from *t* = 20 s to *t* = 320 s.

### Electron microscopy.

Procedures to prepare samples for transmission electron microscopy (TEM) included high-pressure freezing (HPF), freeze substitution, and Epon embedding, which were carried out as described previously (92). Briefly, human fibroblasts were seeded in μ-Slides (Ibidi GmbH, Martinsried, Germany) containing carbon-coated sapphire discs (Engineering Office M. Wohlwend GmbH) 1 day prior to infection at 80% to 90% confluence. Cells were infected with virus overnight at an MOI of 1. Medium containing viral inocula was replaced with fresh medium the next day. Infected cells on sapphire discs were fixed by using HPF with a Compact 01 high-pressure freezer (Engineering Office M. Wohlwend GmbH, Sennwald, Switzerland) at 5 dpi. Thereafter, cells on sapphire discs were processed by freeze-substitution and subsequently embedded in Epon (Fluka, Buchs, Switzerland). Ultrathin sections of the Epon-embedded cells were cut with an ultramicrotome (Ultracut UCT; Leica Microsystems, Wetzlar, Germany) and placed on Formvar-coated single-slot grids (Plano GmbH, Wetzlar, Germany). Grids were examined in a Jeol JEM-1400 (Jeol Ltd., Tokyo, Japan) transmission electron microscope equipped with a charge-coupled-device (CCD) camera at an acceleration voltage of 120 kV. Fixation and embedding of infected cells for scanning transmission electron microscopy (STEM) tomography was the same as described for TEM. Additional sample preparation steps were conducted as described previously (93, 94). Tomogram acquisition was conducted on a STEM Jeol JEM-2100F with an acceleration voltage of 200 kV. Tilt series were acquired from 600-nm-thin sections from +72° to −72° with a 1.5° increment using the bright field detector. Image series were reconstructed to tomograms by weighted back projection with the IMOD software package (95). 3D visualization of the membrane structures was performed using Avizo lite software (Visualization Science Group, Burlington, MA, USA) by threshold segmentation.

### Western blotting.

Western blotting procedures, including the primary and secondary antibodies used for the detection of the HA tag, UL148, ATF4, eIF2α, eIF2α-P (Ser51), the HCMV viral nuclear antigen IE1, and the conditions used for the detection of Ser51 phosphorylated eIF2α, were carried out as described previously (8, 10). Additional antibodies used in this study were mouse anti-glyceraldehyde-3-phosphate dehydrogenase (GAPDH) (catalog number 60004-1; Proteintech, Rosemont, IL,), mouse anti-gB clone 27-180 (96) (a generous gift from William J. Britt), and anti-GFP (D5.1) XP rabbit monoclonal antibody (MAb) 2956 (Cell Signaling Technology, Danvers, MA).

### Solubility analyses.

For the experiments shown in Fig. 8, 2.0 × 10^5^ human fibroblasts were infected at an MOI of 1 TCID_50_ per cell with TB_148^HA^ or TB_159^HA^. The following day, cells were washed twice with PBS to remove viral inoculum and replenished with DMEM containing 5% newborn calf serum. At various times postinfection, cells were washed once with PBS and lysed by direct addition of 100 μl RIPA buffer (25 mM HEPES [pH 7.5], 400 mM NaCl, 0.1% SDS, 0.5% sodium deoxycholate, 1% NP-40, supplemented with 1× protease inhibitor cocktail [APExBio]). Lysates were collected and rotated at 4°C for 1 h. Insoluble material was pelleted by centrifugation (21,000 × *g*, 35 min). Supernatants containing soluble material were transferred to a fresh microcentrifuge tube, and 33 μl of 4× Laemmli sample buffer (200 mM Tris [pH 6.8], 8% SDS, 40% glycerol, and 0.08% bromophenol blue) was added to bring the final volume to 133 μl. The pellet was disrupted in 133 μl of 1× Laemmli buffer prepared by diluting 4× Laemmli buffer in RIPA buffer. Samples were reduced by the addition of 5% final (vol/vol) beta-mercaptoethanol (BME) and boiled at 90°C for 10 min. Forty microliters of each sample was resolved by SDS-PAGE (12% polyacrylamide gel), transferred overnight to a nitrocellulose membrane, and immunoblotted with antibodies against the HA epitope or HCMV gB. Quantitation of secondary antibody fluorescence signals was performed using an Odyssey CLx scanner (LI-COR, Inc., Lincoln, NE) in auto-scan mode. For each time point, the signals from RIPA buffer-soluble and -insoluble bands were summed to yield total signal, and the ratio of insoluble band signal to total signal was also calculated. For the experiments shown in Fig. S4D, i148^HA^ ARPE-19 cells were seeded into 6-well plates at 1 × 10^6^ cells per well and incubated overnight (5% CO_2_, 37°C) in Opti-MEM medium supplemented with 2% tet-approved FBS (Clontech), 20 μg/ml gentamicin sulfate, 10 μg/ml ciprofloxacin HCl, and 1 μg/ml puromycin HCl. The following day, fresh Opti-MEM supplemented as above but additionally including 100 ng/ml dox was applied to induce UL148^HA^. Twenty-four hours later, the cells were washed twice in PBS (catalog number 21-040-CV; Corning) to remove dox, and then fresh Opti-MEM was supplemented as described above with tet-approved FBS, gentamicin, and ciprofloxacin but also containing either 10 μM epoxomicin, 115 nM folimycin (added from 1,000× stock solutions), or DMSO vehicle alone (0.1% [vol/vol]). A time course series of samples was harvested, the first immediately after dox washout (0 h), and at 6 h, 12 h, 18 h, and 24 h following the addition of epoxomicin, folimycin, or DMSO vehicle control. At various times posttreatment, the medium was removed, the cells were washed in PBS and then scraped into 150 μl RIPA lysis buffer. Collected lysates were then spun at 21,000 × *g* for 30 min at 4°C. Supernatant fractions were transferred to fresh tubes, mixed with 50 μl of 4× Laemmli sample buffer containing 5% BME, and heated at 95°C for 10 min. Pellets containing RIPA buffer-insoluble material were each vortexed in 150 μl RIPA buffer supplemented with 50 μl of Laemmli sample buffer containing 5% BME and heated at 95°C for 10 min. Twenty-five microliters of each sample was then resolved on 12% acrylamide SDS-PAGE gels, transferred to nitrocellulose membranes (0.45-μm pore size), stained briefly using Ponceau S, and immunoblotted for the various proteins.

### Statistical analyses.

Statistical analyses were carried out using GraphPad Prism 8.1.0 for MacOS (GraphPad, Inc., San Diego, CA).

10.1128/mBio.02110-19.9 MOVIE S5 i148^GFP^ cells, 200 nM ISRIB, 2 to 19 h post-dox induction. Download Movie S5, MPG file, 11.4 MB. Copyright © 2019 Zhang et al. 2019 Zhang et al. This content is distributed under the terms of the Creative Commons Attribution 4.0 International license.
