# Supplementary figures and images for: The Human Cytomegalovirus Nonstructural Glycoprotein UL148 Reorganizes the Endoplasmic Reticulum
Source: mBio. 2019 Dec 10;10(6):e02110-19. doi: 10.1128/mBio.02110-19 (PMC6904874; doi:10.1128/mBio.02110-19)

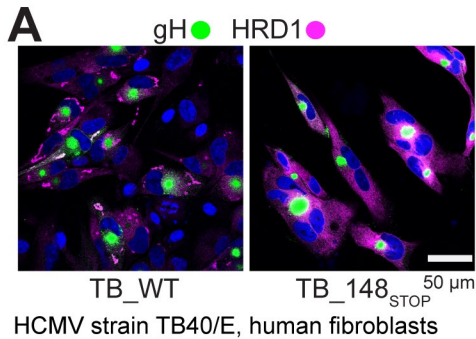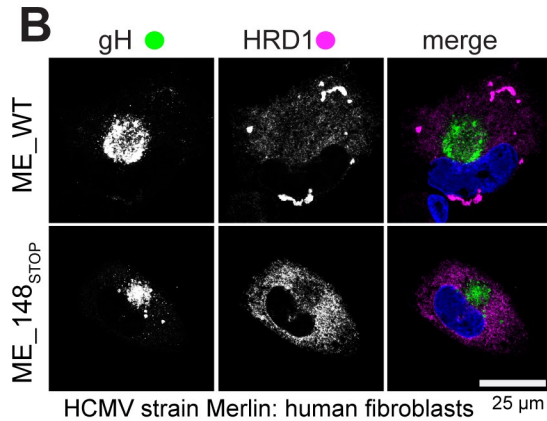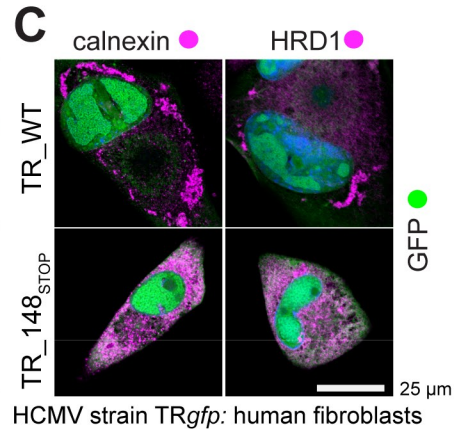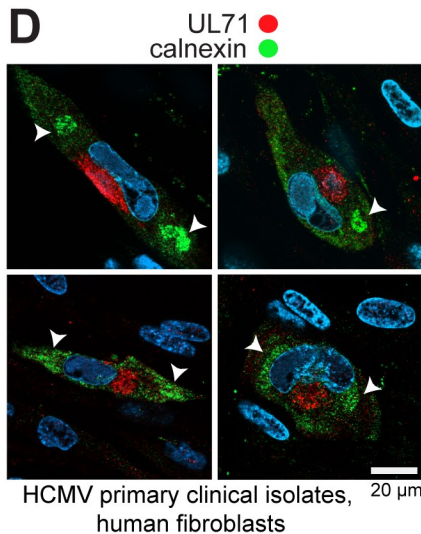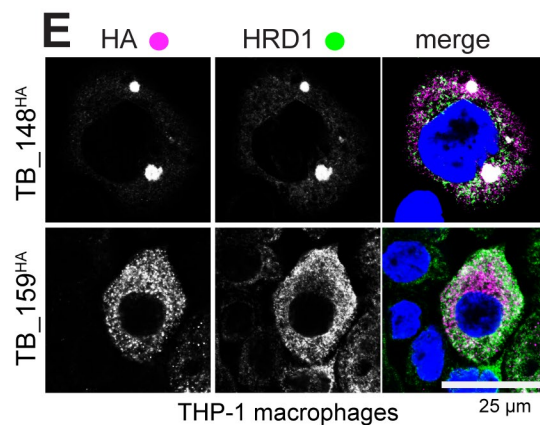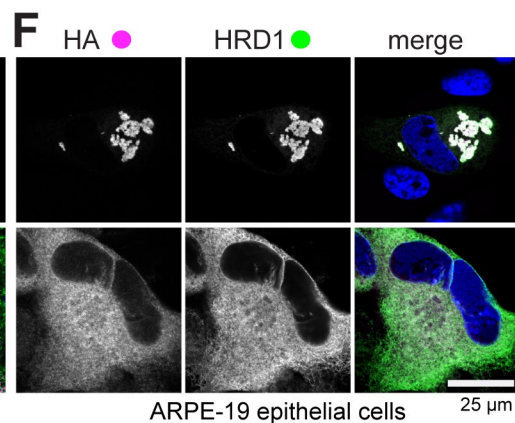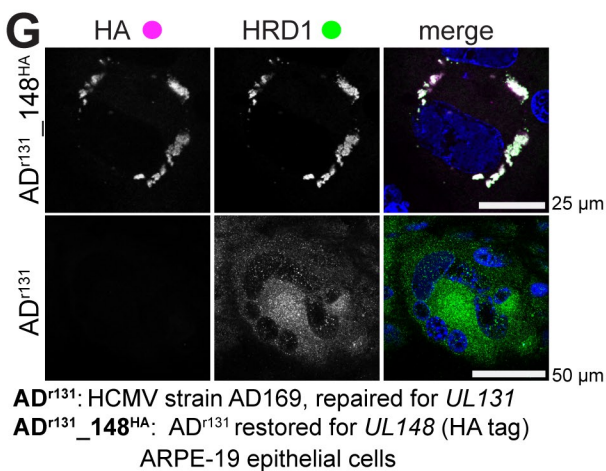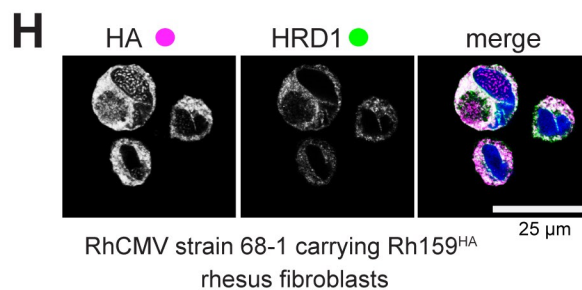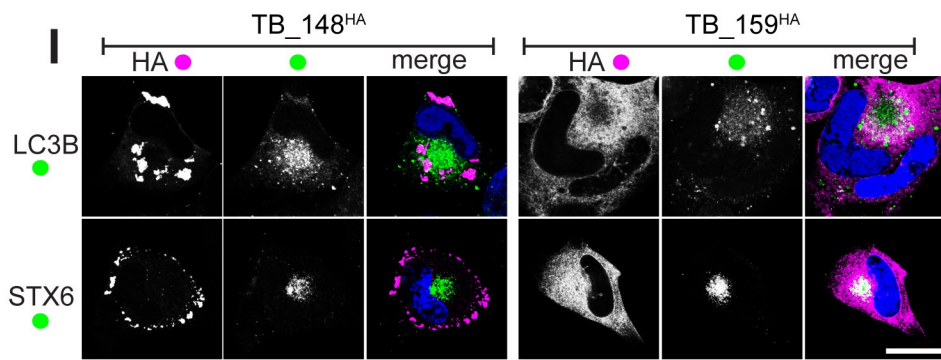

Supplement: FIG S1 [file mBio.02110-19-sf001.pdf]

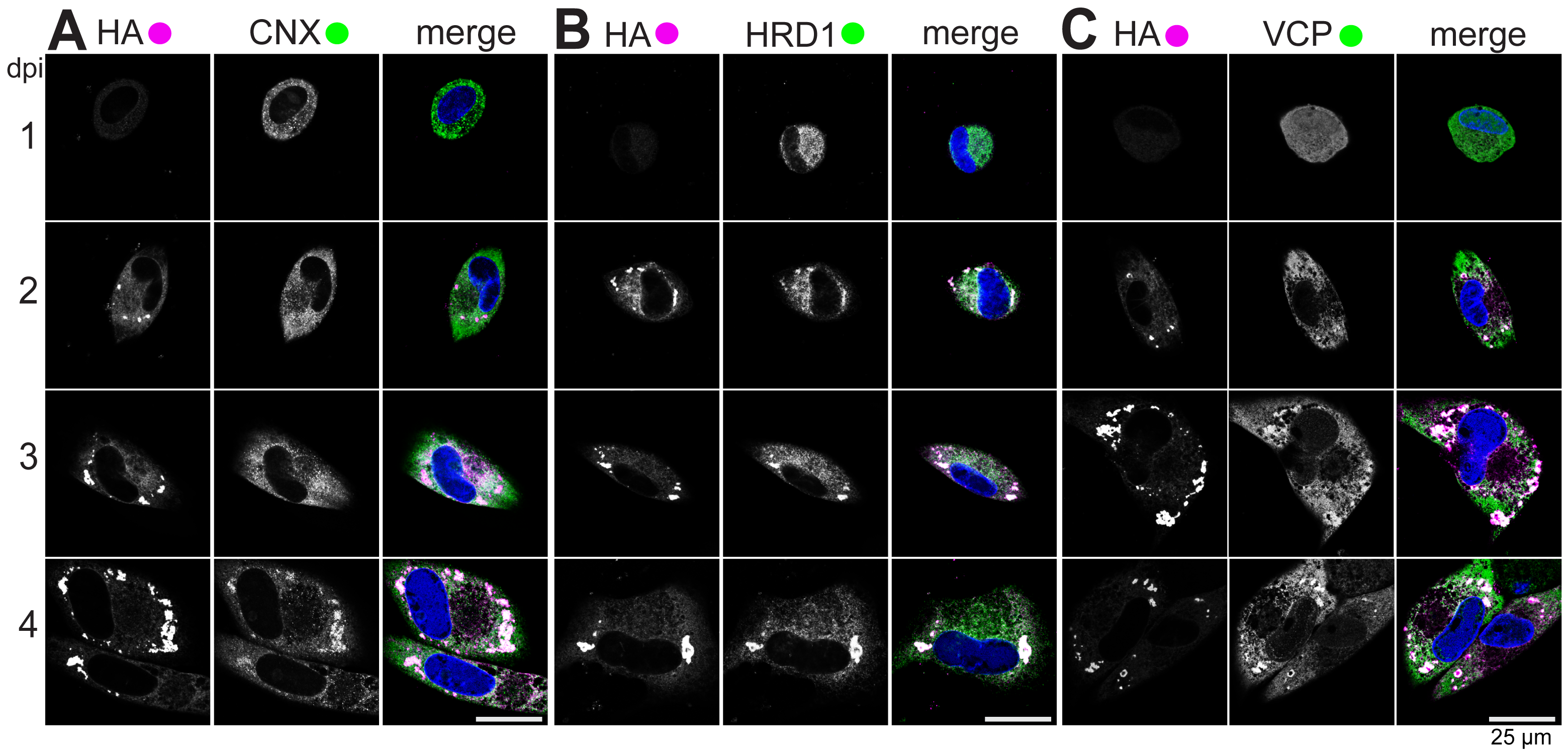

Supplement: FIG S2 [file mBio.02110-19-sf002.tif]

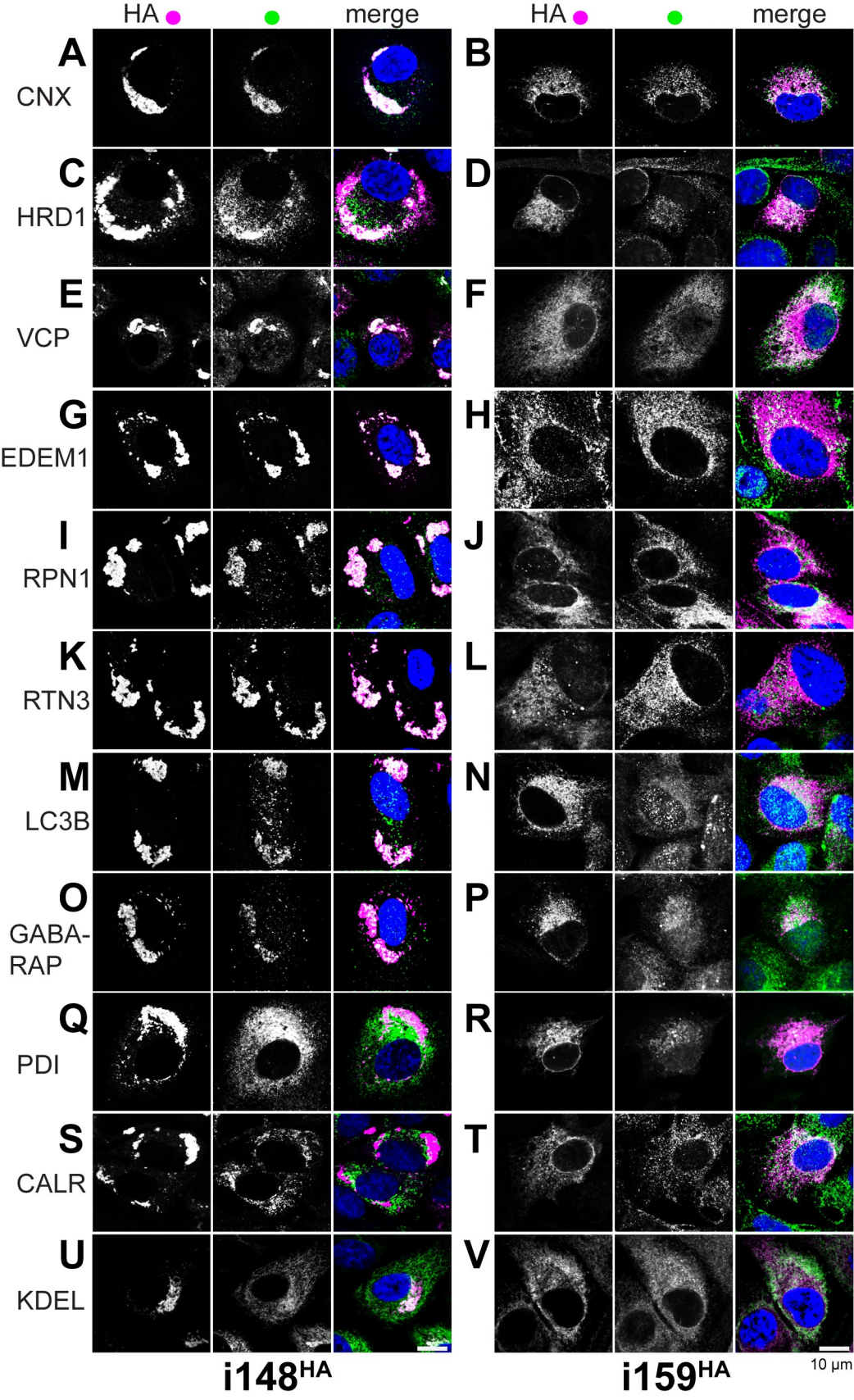

Supplement: FIG S3 [file mBio.02110-19-sf003.pdf]

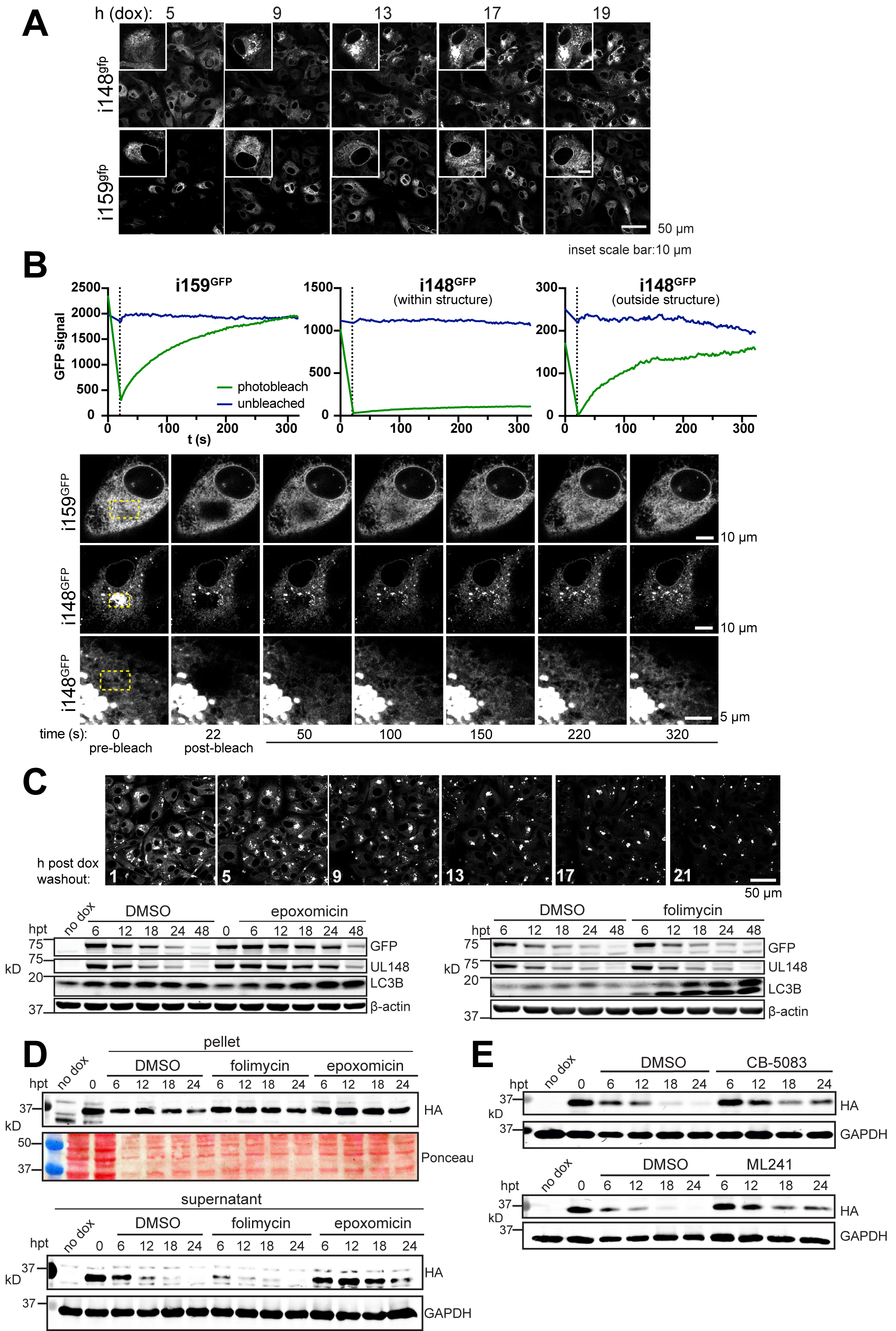

Supplement: FIG S4 [file mBio.02110-19-sf004.tif]
