# Supplementary material for: The Human Cytomegalovirus Nonstructural Glycoprotein UL148 Reorganizes the Endoplasmic Reticulum
Source: mBio. 2019 Dec 10;10(6):e02110-19. doi: 10.1128/mBio.02110-19 (PMC6904874; doi:10.1128/mBio.02110-19)
Supplement: TABLE S1 [file mBio.02110-19-st001.docx]

| **TABLE S1A. Oligonucleotide primers and synthetic DNAs used in this study** | |
| --- | --- |
| **Name** | **Sequence (5' to 3')** |
| 148_eGFP_Fw | ﻿ACCTGGTGTCGCGGCGTCGGATGGTGAGCAAGGGGGAGGAGCTTTTTACTGGCGTTGTTC |
| Gibs_eGFP_Rv | ﻿CGGCCGCCACTGTGCTGGATTACTTATACAGCTCGTCCATACCCAG |
| Gib_148_Fw | ﻿TGTGGTGGAATTCTGCAGATTGCCACCATGTTGCGCTTGCTG |
| 148_noStopRv | ﻿CCGACGCCGCGACACCAGGTAGGTTATC |
| 159_eGFP_Fw | TCTAAAACGCAGTCGTGAAGCTCATATGGTGAGCAAGGGGGAGGA |
| 159_noStop_Rv | ATGAGCTTCACGACTGCGTTTTAGAAGTG |
| Gibs_159_Fw | TGTGGTGGAATTCTGCAGATTGCCACCATGGCCTACAACAGC |
| Rh159_Fw | ﻿TGTGGTGGAATTCTGCAGATACCATGGCCTACAACAGCTTCCTTCTCAGCTGCCTTACCATCGCACTTTTACTG |
| Rh159_HA_Rv | CGGCCGCCACTGTGCTGGATCTATTAGGCGTAGTCTGGGACGTCGTATGGGTAATGAGCTTCACGACTGCGTTTTAGAAGTG |
| PpuISceKanGibs_Fw | CATTCACCTGAACCCATAGGTCCTGCCTGGCAGAGAATCTTAGGGATAACAGGGTAATCG |
| PpuISceKanGibs _Rv | AGATTCTCTGCCAGGCAGGACCTATGGGTTCAGGTGAATGGCCAGTGTTACAACCAATTA |
| Rh159_Fw_recomb | GGGCGGTGGCCGGCACGCCGCATTTCCTAACCCGCGCAGCATGGCCTACAACAGCTTCCT |
| Rh159_Rv_recomb | CGCGAACGACGTGTGACGAGGACGTGGTTTCCGCAAGCCTTTAGGCGTAGTCTGGGACG |
| TB_159HA_seq_Fw | AGTGTTTATGAGTCGGGCGG |
| TB_159HA_seq_R | GTGGCGACGTGGATTTCTTG |
| EGFP-P2A-3XHA | ﻿GTCGACTGGATCCGGTACCGAATTCGCCACCATGGTGAGCAAGGGGGAGGAGCTTTTTACTGGCGTTGTTCCCATCCTGGTTGAGTTGGATGGGGATGTCAACGGCCACAAGTTCAGCGTTTCTGGAGAAGGAGAAGGCGACGCAACCTATGGGAAGCTCACTTTGAAATTCATATGCACCACCGGTAAACTCCCAGTCCCCTGGCCTACCCTGGTCACTACCTTGACATATGGAGTTCAGTGCTTTTCTCGATACCCCGACCACATGAAGCAGCACGATTTTTTCAAATCTGCAATGCCCGAGGGCTATGTACAAGAGAGGACCATTTTCTTCAAAGACGACGGCAATTACAAGACCAGGGCCGAGGTCAAATTTGAAGGGGACACTCTTGTGAATCGGATCGAACTTAAAGGGATCGATTTCAAAGAGGACGGAAACATACTGGGGCATAAGCTGGAATACAACTACAATAGCCATAATGTTTACATTATGGCAGACAAGCAGAAAAACGGTATTAAGGTCAACTTCAAGATACGGCATAACATTGAAGATGGGTCAGTCCAGCTTGCTGACCATTATCAGCAGAATACACCCATTGGCGATGGTCCCGTGCTTTTGCCTGACAACCACTACCTTTCTACCCAAAGCGCATTGTCAAAAGATCCTAACGAGAAGAGAGATCATATGGTCCTTTTGGAGTTCGTAACCGCAGCTGGTATAACTCTGGGTATGGACGAGCTGTATAAGGGCTCAGGGGCTACAAATTTTAGTCTCCTTAAACAAGCCGGAGATGTCGAAGAGAACCCTGGGCCTTACCCATATGACGTTCCTGACTATGCCGGTTACCCCTATGATGTGCCAGATTATGCCGGATCTTATCCTTACGATGTACCTGACTATGCAGGTGGGAGTGGCATGAATTACGTGGGGCAGC |
| Gibs_Age_159_Fw | CCGTACCACTTCCTACCCTCGTAAACCGGTGCCACCATGGCCTACAACAG |
| Gibs_Mlu_GFP_Rv | ACCCGGCGCGGAGGCCAGATCTTAACGCGTTTACTTATACAGCTCGTCCATACC |
| tRFP linker Fw | ﻿CCACTTCCTACCCTCGTAAACCGGTGAATTCGCCACCATGAGCGAGCTGAT |
| tRFP linker Rv | ﻿GCGCGGAGGCCAGATCTTAACGCGTGCGGCCGCTCGAGAATTCATCTGTGCCCCAGTTTGC |

**TABLE S1B. Antibodies Used in Immunofluorescence Microscopy**

| **Primary Antibody** | **Host species** | **Isotype** | **Clone** | **Vendor** | **Catalog No.** | **Dilution used** |
| --- | --- | --- | --- | --- | --- | --- |
| Calnexin | Mouse | mAb IgG1 | 37 | BD Biosciences | 610523 | 1:50 |
| Calnexin | Rabbit | mAb IgG | C5C9 | Cell Signaling Tech. | 2679 | 1:50 |
| Syntaxin 6 | Rabbit | mAb IgG | C34B2 | Cell Signaling Tech. | 2869 | 1:50 |
| SYVN1 (Hrd1) | Rabbit | mAb IgG | D3O2A | Cell Signaling Tech. | 14773 | 1:500 |
| SEL1L | Rabbit | pAb | n/a | Sigma | S3699 | 1:50 |
| KDEL | Mouse | IgG2a | 10C3 | Enzo Life Sci., Inc. | ADI-SPA-827-D | 1:50 |
| PDI | Rabbit | mAb IgG | C81H6 | Cell Signaling Tech. | 3501 | 1:50 |
| Herp (HerpUD1) | Rabbit | pAb IgG | n/a | Proteintech | 10813-1-AP | 1:50 |
| VCP | Mouse | IgG2a | 5 | Invitrogen / Life Technologies | MA3-004 | 1:50 |
| Calreticulin | Rabbit | mAb IgG | D3E6 | Cell Signaling Tech. | 12238 | 1:200 |
| Ribophorin 1 (RPN1) | Rabbit | pAb IgG |  | ThermoFisher / Invitrogen | PA5-65662 | 1:50 |
| EDEM-1 | Mouse | mAb IgM | D-1 | Santa Cruz | sc-377394 | 1:50 |
| GABARAP | Rabbit | mAb | E1J4E | Cell Signaling Tech. | 13733 | 1:200 |
| LC3B | Rabbit | mAb | D11 | Cell Signaling Tech. | 3868 | 1:200 |
| HA epitope | Chicken | IgY |  | Bethyl | A190-106A | 1:200 |
| HCMV glycoprotein H (gH) | Mouse | IgG2b | 14-4b | Gift of William J. Britt, M.D. | n/a | 1: 50 |
| Reticulon-3 (RTN3) | Mouse | IgG2a |  | ThermoFisher / Invitrogen | MA5-15538 | 1: 200 |
| HA epitope | Rabbit | pAb |  | Bethyl | A190-108A | 1:1000 |
|  |  |  |  |  |  |  |
| **Secondary Antibody** | **Host species** | **Isotype** |  | **Vendor** | **Catalog No.** | **Dilution used** |
| Alexa Fluor 488 anti-Mouse IgG (H+L), cross adsorbed | Goat | IgG |  | ThermoFisher / Invitrogen | A11001 | 1:1000 |
| Alexa Fluor 488 anti-Rabbit IgG (H+L) Cross-Adsorbed | Goat | IgG |  | ThermoFisher / Invitrogen | A11008 | 1:1000 |
| Alexa Fluor 594 anti-Rabbit IgG (H+L) Cross-Adsorbed | Goat | IgG |  | ThermoFisher / Invitrogen | A11012 | 1:1000 |
| Alexa Fluor 594 anti-Mouse IgG (H+L) Cross-Adsorbed | Goat | IgG |  | ThermoFisher / Invitrogen | A11005 | 1:1000 |
| Alexa Fluor 647 anti-Chicken IgY (H+L) | Goat | IgG |  | ThermoFisher / Invitrogen | A11039 | 1:1000 |
